# Supplementary material for: Anomalous deep-red luminescence of perylene black analogues with strong π-π interactions
Source: Nat Commun. 2023 Apr 6;14:1922. doi: 10.1038/s41467-023-37171-y (PMC10079835; doi:10.1038/s41467-023-37171-y)
Supplement: Supplementary file 1 — Supplementary Information [file 41467_2023_37171_MOESM1_ESM.pdf]

Supplementary Information

Anomalous Deep-Red Luminescence of Perylene Black Analogues with Strong π-π Interactions

Ningning Tang[1], Jiadong Zhou*[,1], Liangxuan Wang,[2,3] Matthias Stolte[4], Guojing Xie[1], Xinbo Wen[1], Linlin Liu[1], Frank Würthner *[,4], Johannes Gierschner*[,2], Zengqi Xie*[,1]

[1] State Key Laboratory of Luminescent Materials and Devices, Institute of Polymer Optoelectronic Materials and Devices, Guangdong Provincial Key Laboratory of Luminescence from Molecular Aggregates, South China University of Technology, Guangzhou 510640, P. R. China.

[2] Madrid Institute for Advanced Studies, IMDEA Nanoscience, Ciudad Universitaria de Cantoblanco, C/ Faraday 9, 28049 Madrid, Spain.

[3] Institute of Physical and Theoretical Chemistry, Eberhard Karls University Tübingen, 72076 Tübingen, Germany

[4] Institut für Organische Chemie and Center for Nanosystems Chemistry, Julius-Maximilians-Universität Würzburg, Am Hubland, 97074 Würzburg (Germany)

*Emails: zhoujd@scut.edu.cn (JZ), wuerthner@uni-wuerzburg.de (FW), johannes.gierschner@imdea.org (JG), msxiez@scut.edu.cn (ZX)

CONTENTS

1. **Experimental Section** S3
2. **Synthesis** S4
3. **NMR Spectra** S9
4. **Single Crystal X-ray Analysis** S20
5. **Photophysical Properties of Monomers** S23
6. **Photophysical Properties of the Thin Films** S30
7. **Ab Initio Molecular Dynamics (AIMD)** S34
8. **DFT Calculation** S36
9. **Supplementary References** S43

1. Experimental Section

The growth of single crystals for structure evaluation. The single crystals of PBIs were successfully obtained by liquid-liquid diffusion method of ethanol into chloroform. For different length of the alkoxyl chains in *N*-position the requirements for the ratio of ethanol and chloroform vary slightly. The **4,6-ab-PBI** crystals were got by dissolving 2 mg **4,6-ab-PBI** powder in 1 mL chloroform solution and filtered using 0.22 μm filters to give a red transparent solution. Then 1.2 mL ethanol was injected slowly with a syringe. The solution was placed for 3-5 days to obtain little flaky red **4,6-ab-PBI** crystals at the bottom of glass vial. As for **6,8-ab-PBI** and **8,10-ab-PBI**, the ratio of ethanol and chloroform were 1:1.4 and 1:1.5 respectively.

The fabrication of crystalline films for experiment. All the crystalline films were fabricated by spin-coated (2500 rpm, 30s) from solution (chloroform for **4,6-ab-PBI** and **6,8-ab-PBI**, chlorobenzene for **8,10-ab-PBI** 15 mg/ml) onto substrates and heat treatment were carried out in air (**4,6-ab-PBI** and **6,8-ab-PBI** were annealed at 180 °C for 30 min, **8,10-ab-PBI** was annealed at 160 °C). The film thickness was measured to be ~30 nm using profilometry (Stylus profiler Dektak XTL, Bruker). The crystallinity of the spin-coated thin films was improved by post annealing treatment under ambient environment. The absorbance of all films at the excitation was under 0.1 for the fluorescence measurements.

Monomers spectral properties at different polarities. All samples were prepared from solvents of different polarities with the concentration of 10^{-5} M for UV/Vis spectra and the maximum absorbance of each sample was under 1. The fluorescence spectra were carried out with the concentration of 10^{-7} M and the excitation wavelength for each sample was 490 nm. After fluorescence spectra measurement, the samples were used for the measurement of lifetime and absolute photoluminescence quantum yield (PLQY). All the data of lifetime were fitted using double exponential fluorescence decay to give a shorter lifetime as well as a longer lifetime.

Temperature-dependent spectra. Data points were collected every 10 K and kept at constant temperature for 5 minutes before recording. For PBIs chloroform solution ($c = 1 \times 10^{-7}$ M), both heating and cooling processes were measured, giving the same results.

2. Synthesis

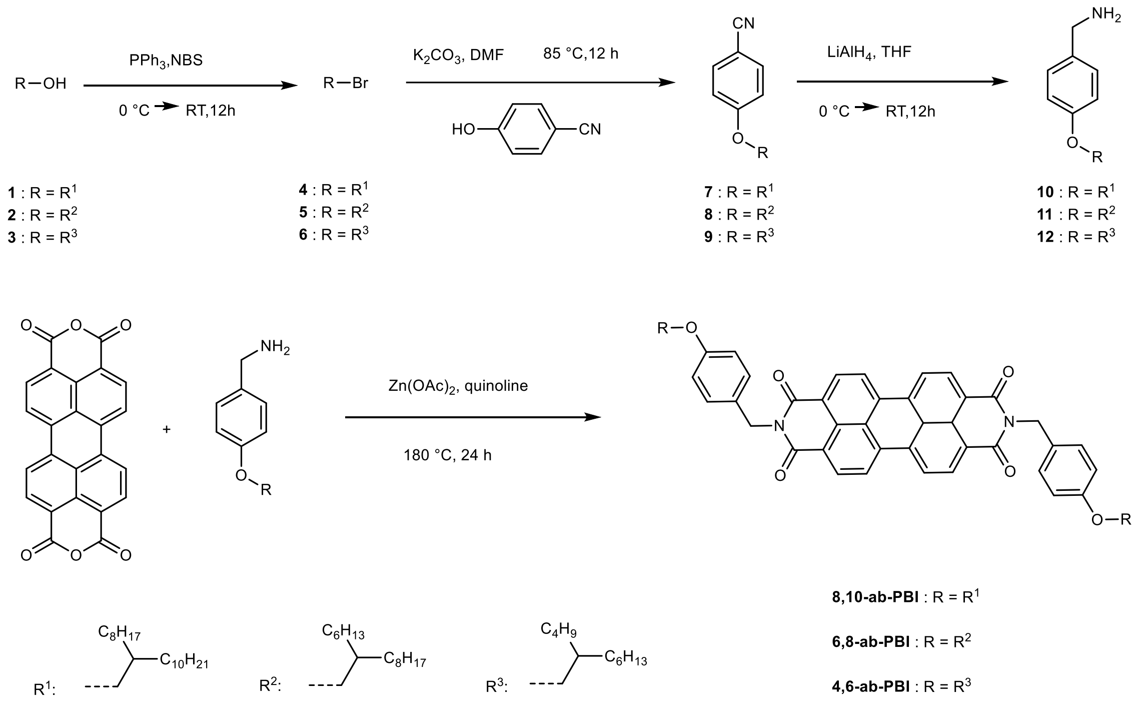

Supplementary Fig. 1 Synthesis route of *N*-alkoxybenzyl substituted PBI derivatives.

Compound 4: To a solution of compound **1** (5.00 g, 16.760 mmol, purity: 97%) in dichloromethane (100 mL, Chemically Pure) was added triphenylphosphine (6.15 g, 23.460 mmol, purity: ≥ 99%) and stirred at 0°C. The mixed solution was added *N*-bromosuccinimide (3.88 g, 21.790 mmol, purity: ≥ 99%) slowly. The mixture was stirred for 12 h at room temperature. The mixture was concentrated under reduced pressure to give a pale-yellow solid. 200 mL petroleum ether was added and consecutively the ultrasonic shaking was applied to extract crude product. Solvent removal and purification by column chromatography (silica gel, petroleum ether) afforded compound 1 (5.43g, 90%) as colorless transparent oily liquid. ^{1}H NMR (500 MHz, $CDCl_3$, 298 K, TMS) δ 3.48-3.46 (d, 2H), 1.65-1.59 (m, 1H), 1.42-1.29 (m, 32H), 0.92-0.89 (t, 6H).

Compound 5: To a solution of compound **2** (5.00 g, 20.639 mmol, purity: 97%) in dichloromethane (100 mL, Chemically Pure) was added triphenylphosphine (7.57 g, 28.890 mmol, purity: ≥ 99%) and stirred at 0°C. The mixed solution was added *N*-bromosuccinimide (4.78 g, 26.831 mmol, purity: ≥ 99%) slowly. The mixture was stirred for 12 h at room temperature. The mixture was concentrated under reduced pressure to give a pale-yellow solid. 200 mL petroleum ether was added and consecutively the ultrasonic shaking was applied to extract crude product. Solvent removal and purification by column chromatography (silica gel, petroleum ether) afforded compound **5** (5.36g, 85%) as colorless transparent oily liquid.^{1}H NMR (500 MHz, $CDCl_3$, 298 K, TMS) δ 3.45-3.44 (d, 2H), 1.62-1.54 (m, 1H), 1.39-1.27 (m, 24H), 0.90-0.87 (m,6H).

Compound 6: To a solution of compound **3** (5.00 g, 26.853 mmol, purity: 97%) in dichloromethane (100 mL, Chemically Pure) was added triphenylphosphine (9.85 g, 37.588 mmol, purity: ≥ 99%) and stirred at 0°C. The mixed solution was added *N*-bromosuccinimide (6.22 g, 34.912 mmol, purity: ≥ 99%) slowly. The mixture was stirred for 12 h at room temperature. The mixture was concentrated under reduced pressure to give a pale-yellow solid. 200 mL petroleum ether was added and consecutively the ultrasonic shaking was applied to extract crude product. Solvent removal and purification by column chromatography (silica gel, petroleum ether) afforded compound **5** (6.07g, 91%) as colorless transparent oily liquid.^{1}H NMR (500 MHz, $CDCl_3$, 298 K, TMS) δ 3.45-3.44 (d, 2H), 1.62-1.56(m, 1H), 1.37-1.27(m, 16H), 0.92-0.87 (m, 6H).

Compound 7: To a 250 mL round-bottom flask 4-hydroxybenzonitrile (1.00 g, 8.390 mmol, purity: 98%) and 100 mL *N*, *N*-dimethylformamide (Analytical reagent) were added, then the mixture was stirred to have a completely dissolved 4-hydroxybenzonitrile solution. Compound **4** (4.50 g, 12.450 mmol) and potassium carbonate (5.80 g, 41.950 mmol, purity: 99%) were added, respectively. The mixture was stirred at 0 °C under nitrogen for 12 h. Afterward ethyl acetate was added and the solution was separated by filtration. The solution was washed three times with saturated salt water and evaporated. The crude product was purified by column chromatography on silica gel using petroleum ether/CH_2Cl_2 (1:3 v/v) as an eluent to give 2.38 g (yield 71%) of a colorless and transparent oil. ^{1}H NMR (500 MHz, $CDCl_3$, 298 K, TMS) δ 7.58-7.56 (d, 2H), 6.94-6.92 (d, 2H), 3.87-3.86 (d, 2H), 1.81-1.76 (m, 1H), 1.45-1.41 (m, 4H), 1.39-1.26 (m, 28H), 0.89-0.86 (t, 6H). ^{13}C NMR (101 MHz, $CDCl_3$) δ 162.71, 133.93, 119.35, 115.23, 108.34, 103.62, 71.31, 39.69, 39.54, 37.81, 36.12, 32.60, 31.94, 31.92, 31.27, 29.98, 29.81, 29.66, 29.62, 29.57, 29.49, 29.37, 29.33, 27.87, 26.82, 26.58, 22.70, 14.12. MALDI-TOF(m/s): calcd. for $C_{27}H_{45}NO$: 399.66; found, 399.34.

Compound 8: To a 250 mL round-bottom flask 4-hydroxybenzonitrile (1.00 g, 8.390 mmol, purity: 98%) and 100 mL *N*, *N*-dimethylformamide (Analytical reagent) were added, then the mixture was stirred to have a completely dissolved 4-hydroxybenzonitrile solution. Compound **5** (3.80 g, 12.445 mmol) and potassium carbonate (5.80 g, 41.950 mmol, purity: 99%) were added, respectively. The mixture was stirred at 0 °C under nitrogen for 12 h. Afterward ethyl acetate was added and the solution was separated by filtration. The solution was washed three times with saturated salt water and evaporated. The crude product was purified by column chromatography on silica gel using petroleum ether/CH_2Cl_2 (1:3 v/v) as an eluent to give 3.42 g (yield 71%) of a colorless and transparent oil.^{1}H NMR (500 MHz, $CDCl_3$, 298 K, TMS) δ 7.59-7.57 (d, 2H), 6.96-6.94 (d, 2H), 3.90-3.88 (d, 2H), 1.84-1.78 (m, 1H), 1.37-1.29 (m, 24H), 0.92-0.88 (m, 6H). ^{13}C NMR (101 MHz, $CDCl_3$) δ 162.70, 133.90, 119.31, 115.22, 108.35, 103.60, 71.29, 37.81, 36.10, 31.92, 31.90, 31.83, 31.28, 31.26, 29.97, 29.64, 29.56, 29.48, 29.14, 27.85, 26.81, 22.68, 14.10. MALDI-TOF(m/s): calcd. for $C_{23}H_{37}NO$: 343.56; found, 344.28.

Compound 9: To a 250 mL round-bottom flask 4-hydroxybenzonitrile (1.00 g, 8.390 mmol, purity: 98%) and 100 mL *N*, *N*-dimethylformamide (Analytical reagent) were added, then the mixture was stirred to have a completely dissolved 4-hydroxybenzonitrile solution. Compound **6** (3.10 g, 12.445 mmol) and potassium carbonate (5.80 g, 41.950 mmol, purity: 99%) were added, respectively. The mixture was stirred at 0 °C under nitrogen for 12 h. Afterward ethyl acetate was added and the solution was separated by filtration. The solution was washed three times with saturated salt water and evaporated. The crude product was purified by column chromatography on silica gel using petroleum ether/CH_2Cl_2 (1:3 v/v) as an eluent to give 3.04 g (yield 85%) of a colorless and transparent oil.^{1}H NMR (500 MHz, $CDCl_3$, 298 K, TMS) δ 7.59-7.56 (d, 2H), 6.96-6.94 (d, 2H), 3.90-3.88 (d, 2H), 1.84-1.78 (m, 1H), 1.45-1.30 (d, 16H), 0.94-0.88 (m, 6H). ^{13}C NMR (101 MHz, $CDCl_3$) δ 162.70, 133.91, 119.32, 115.22, 108.34, 103.60, 71.29, 37.79, 31.82, 31.28, 30.96, 30.04, 29.63, 29.14, 29.03, 26.79, 23.01, 22.66, 14.09. MALDI-TOF(m/s): calcd. for $C_{19}H_{29}NO$: 287.45; found, 287.17.

Compound 10: To a 250 mL round-bottom flask $LiAlH_4$ (15.000 mmol, purity: 97%) and 30 mL ultra-dry THF (purity: ≥ 99.5%) were added. A solution of compound **7** (2.00 g, 5.000 mmol) in 10 mL THF was added dropwise to the flask at 0 °C under nitrogen. The mixture solution was stirred at ambient temperature for 12 h. After being cooled to 0 °C and diluted with 30 mL ether, the reaction mixture was quenched with water (410 μL), NaOH aqueous solution (410 μL, 15%) and water (1230 μL) and stirred at ambient temperature for 1 h. Afterward the mixture was extracted by ether and washed three times with saturated salt water. Then ether solution was then concentrated under reduced pressure to give a colorless transparent liquid. The crude product was purified by column chromatography on silica gel using CH_2Cl_2 / triethylamine (200:1 v/v) as an eluent to give 1.21 g (yield 60%) of a yellow and transparent oil. ^{1}H NMR (500 MHz, $CDCl_3$, 298 K, TMS) δ 7.22-7.20 (d, 2H), 6.88-6.85 (d, 2H), 3.82-3.81 (d, 2H), 3.80 (s, 2H), 1.78-1.72 (m, 1H), 1.48 (s, 2H), 1.46-1.26 (m, 32H), 0.90-0.86 (t, 6H). ^{13}C NMR (126 MHz, $CDCl_3$) δ 157.50, 127.36, 127.34, 113.60, 113.53, 113.49, 69.97, 44.56, 36.93, 36.44, 32.47, 30.90, 30.89, 30.34, 29.41, 29.01, 28.65, 28.62, 28.57, 28.33, 28.31, 27.95, 27.67, 25.81, 25.38, 21.67, 13.10. MALDI-TOF(m/s): calcd. for $C_{27}H_{49}NO$: 403.70; found, 403.28.

Compound 11: To a 250 mL round-bottom flask $LiAlH_4$ (15.000 mmol, purity: 97%) and 30 mL ultra-dry THF (purity: ≥ 99.5%) were added. A solution of compound **8** (1.72 g, 5.000 mmol) in 10 mL THF was added dropwise to the flask at 0 °C under nitrogen. The mixture solution was stirred at ambient temperature for 12 h. After being cooled to 0 °C and diluted with 30 mL ether, the reaction mixture was quenched with water (410 μL), NaOH aqueous solution (410 μL, 15%) and water (1230 μL) and stirred at ambient temperature for 1 h. Afterward the mixture was extracted by ether and washed three times with saturated salt water. Then ether solution was then concentrated under reduced pressure to give a colorless transparent liquid. The crude product was purified by column chromatography on silica gel using CH_2Cl_2

/ triethylamine (200:1 v/v) as an eluent to give 1.48 g (yield 85%) of a yellow and transparent oil. ^{1}H NMR (500 MHz, $CDCl_3$, 298 K, TMS) δ 7.22-7.20 (d, 2H), 6.87-6.86 (d, 2H), 3.82-3.81 (d, 2H), 3.79 (s, 2H), 1.82-1.77 (m, 1H), 1.70-1.69 (d, 2H), 1.35-1.26 (m, 24H), 0.90-0.87 (t, 6H). ^{13}C NMR (126 MHz, $CDCl_3$) δ 157.47, 128.10, 127.78, 127.43, 115.35, 113.51, 69.98, 44.47, 36.94, 30.89, 30.84, 30.35, 30.34, 29.01, 28.99, 28.68, 28.66, 28.57, 28.31, 25.81, 25.79, 21.66, 13.09. MALDI-TOF(m/s): calcd. for $C_{23}H_{41}NO$: 347.59; found, 348.34.

Compound 12: To a 250 mL round-bottom flask $LiAlH_4$ (15.000 mmol, purity: 97%) and 30 mL ultra-dry THF (purity: ≥ 99.5%) were added. A solution of compound **9** (1.44 g, 5.000 mmol) in 10 mL THF was added dropwise to the flask at 0 °C under nitrogen. The mixture solution was stirred at ambient temperature for 12 h. After being cooled to 0 °C and diluted with 30 mL ether, the reaction mixture was quenched with water (410 μL), NaOH aqueous solution (410 μL, 15%) and water (1230 μL) and stirred at ambient temperature for 1 h. Afterward the mixture was extracted by ether and washed three times with saturated salt water. Then ether solution was then concentrated under reduced pressure to give a colorless transparent liquid. The crude product was purified by column chromatography on silica gel using CH_2Cl_2 / triethylamine (200:1 v/v) as an eluent to give 1.24 g (yield 85%) of a yellow and transparent oil. ^{1}H NMR (500 MHz, $CDCl_3$, 298 K, TMS) δ 7.22-7.20 (d, 2H), 6.87-6.86 (d, 2H), 3.82-3.81 (d, 2H), 3.79 (s, 1H), 1.79-1.74 (m, 1H), 1.68 (s, 1H), 1.46-1.28 (m, 16H), 0.91-0.87 (m, 6H). ^{13}C NMR (126 MHz, $CDCl_3$) δ 157.47, 127.85, 127.34, 113.59, 113.49, 113.18, 69.95, 44.61, 36.92, 30.84, 30.36, 30.04, 28.67, 28.34, 28.03, 25.79, 22.04, 21.65, 13.09. MALDI-TOF(m/s): calcd. for $C_{19}H_{33}NO$: 291.48; found, 292.36.

8,10-ab-PBI: To a 100 mL round-bottom flask compound **10** (1.00 g, 2.480 mmol), 3,4,9,10-perylenetetracarboxylic acid anhydride (0.44g,1.130 mmol, purity: 98%), $Zn(OAc)_2$ (0.21 g, 1.130 mmol, Analytical reagent) and 20 mL quinoline were added. The mixture was stirred at 180 °C under nitrogen for 24 h. Afterward the mixture was dropped into 100 mL 2M HCl aqueous solution and stirred for 1 h. The red solid was speparated by filtration and washed with water and methanol. The crude product was purified by column chromatography on silica gel using petroleum ether/CH_2Cl_2 (1:10 v/v) as an eluent to give 870 mg (60%) of a red solid. ^{1}H NMR (500 MHz, $CDCl_3$, 298 K, TMS) δ 8.48-8.46 (d, 4H), 8.25-8.23 (d, 4H), 7.58-7.56 (d, 4H), 6.89-6.86 (d, 4H), 5.31 (s, 4H), 3.79-3.78 (d, 4H), 1.77-1.70 (m, 2H), 1.42-1.22 (d, 64H), 0.87-0.82 (m, 12H). ^{13}C NMR (126 MHz, $CDCl_3$) δ 162.20, 157.99, 133.34, 130.35, 129.79, 128.08, 127.95, 125.09, 122.19, 121.90, 113.38, 69.83, 42.17, 36.89, 31.71, 30.87, 30.85, 30.30, 29.69, 28.98, 28.62, 28.59, 28.54, 28.30, 28.28, 27.67, 26.42, 21.64, 21.62, 13.08, 13.07. MALDI-TOF(m/s): calcd. for $C_{78}H_{104}N_2O_6$: 1165.70; found, 1165.73.

6,8-ab-PBI To a 100 mL round-bottom flask compound **11** (0.86 g, 2.480 mmol), 3,4,9,10-perylenetetracarboxylic acid anhydride (0.44g,1.130 mmol, purity: 98%), $Zn(OAc)_2$ (0.21 g, 1.130 mmol, Analytical reagent) and 20 mL quinoline were added. The mixture was stirred at 180 °C under nitrogen

for 24 h. Afterward the mixture was dropped into 100 mL 2M HCl aqueous solution and stirred for 1 h. The red solid was speparated by filtration and washed with water and methanol. The crude product was purified by column chromatography on silica gel using petroleum ether/CH_2Cl_2 (1:10 v/v) as an eluent to give 880 mg (67%) of a red solid. ^{1}H NMR (500 MHz, $CDCl_3$, 298 K, TMS) δ 8.50-8.48 (d, 4H), 8.32-8.31 (d, 4H), 7.49-7.48 (d, 4H), 6.80-6.78 (d, 4H), 5.25 (s, 4H), 3.72-3.71 (d, 4H), 1.67-1.64 (m, 2H), 1.24-1.15 (m, 48H), 0.79-0.76 (t, 12H). ^{13}C NMR (126 MHz, $CDCl_3$) δ 163.25, 159.01, 134.41, 131.40, 130.81, 129.14, 128.98, 126.15, 123.23, 122.96, 114.41, 70.85, 43.20, 37.92, 31.88, 31.83, 31.33, 30.00, 29.67, 29.56, 29.30, 28.70, 26.81, 26.78, 22.65, 21.88, 14.64, 14.08. MALDI-TOF(m/s): calcd. for $C_{70}H_{88}N_2O_6$: 1053.48; found, 1053.61.

4,6-ab-PBI To a 100 mL round-bottom flask compound **10** (0.72 g, 2.480 mmol), 3,4,9,10-perylenetetracarboxylic acid anhydride (0.44g,1.130 mmol, purity: 98%), $Zn(OAc)_2$ (0.21 g, 1.130 mmol, Analytical reagent) and 20 mL quinoline were added. The mixture was stirred at 180 °C under nitrogen for 24 h. Afterward the mixture was dropped into 100 mL 2M HCl aqueous solution and stirred for 1 h. The red solid was speparated by filtration and washed with water and methanol. The crude product was purified by column chromatography on silica gel using petroleum ether/CH_2Cl_2 (1:10 v/v) as an eluent to give 640 mg (55%) of a red solid. ^{1}H NMR (500 MHz, $CDCl_3$, 298 K, TMS) δ 8.46-8.44 (d, 4H), 8.20-8.18 (d, 4H), 7.61-7.57 (m, 4H), 6.92-6.88 (m, 4H), 5.33 (s, 4H), 3.82- 3.81 (d, 4H), 1.77-1.73, 1.46 (m, 2H), 1.42-1.25 (m, 32H), 0.90-0.85 (m, 12H). ^{13}C NMR (126 MHz, $CDCl_3$) δ 163.19, 159.02, 134.30, 131.76, 130.84, 129.05, 128.99, 126.04, 123.19, 122.89, 114.42, 70.85, 43.19, 37.91, 31.83, 31.34, 31.03, 29.67, 29.03, 26.79, 23.04, 22.65, 14.08, 14.07. MALDI-TOF(m/s): calcd. for $C_{62}H_{72}N_2O_6$: 941.27; found, 941.47.

3. NMR Spectra

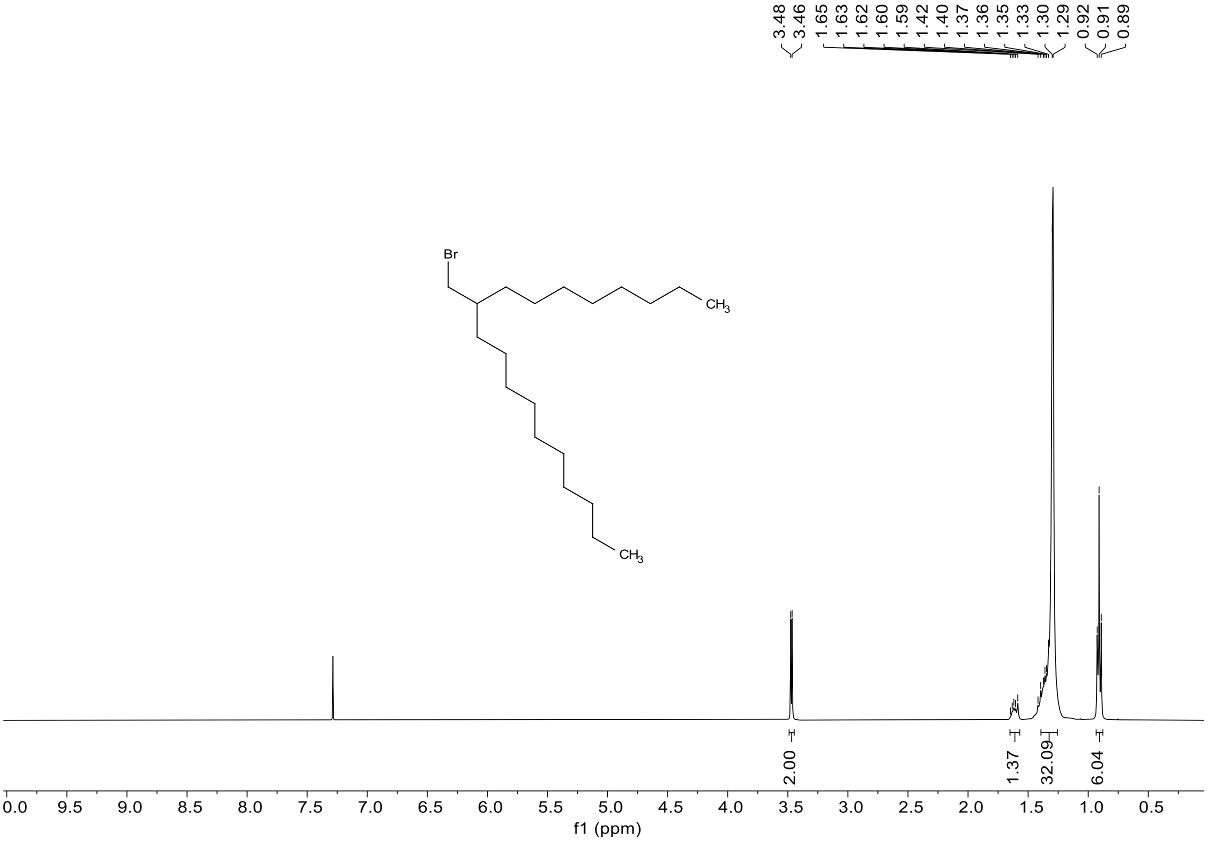

Supplementary Fig. 2 | ^{1}H NMR of **compound 4** (500 MHz, $CDCl_3$, 298 K).

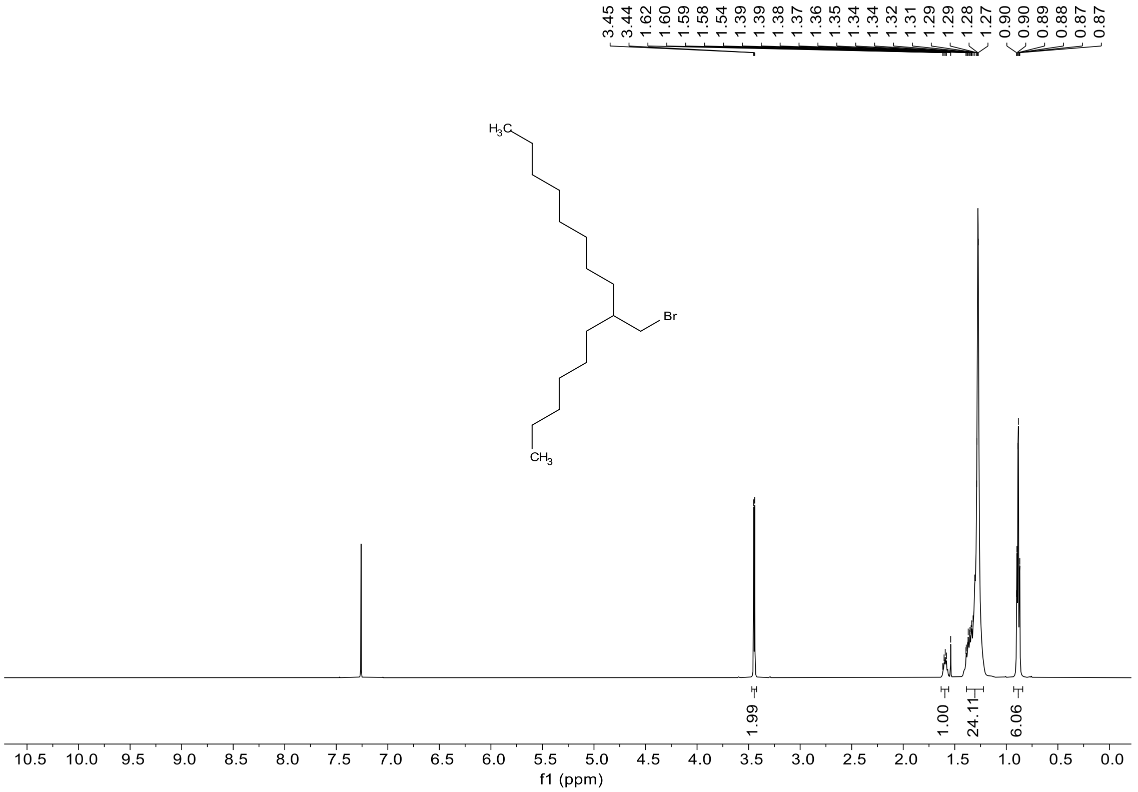

Supplementary Fig. 3 | ^{1}H NMR of **compound 5** (500 MHz, $CDCl_3$, 298 K).

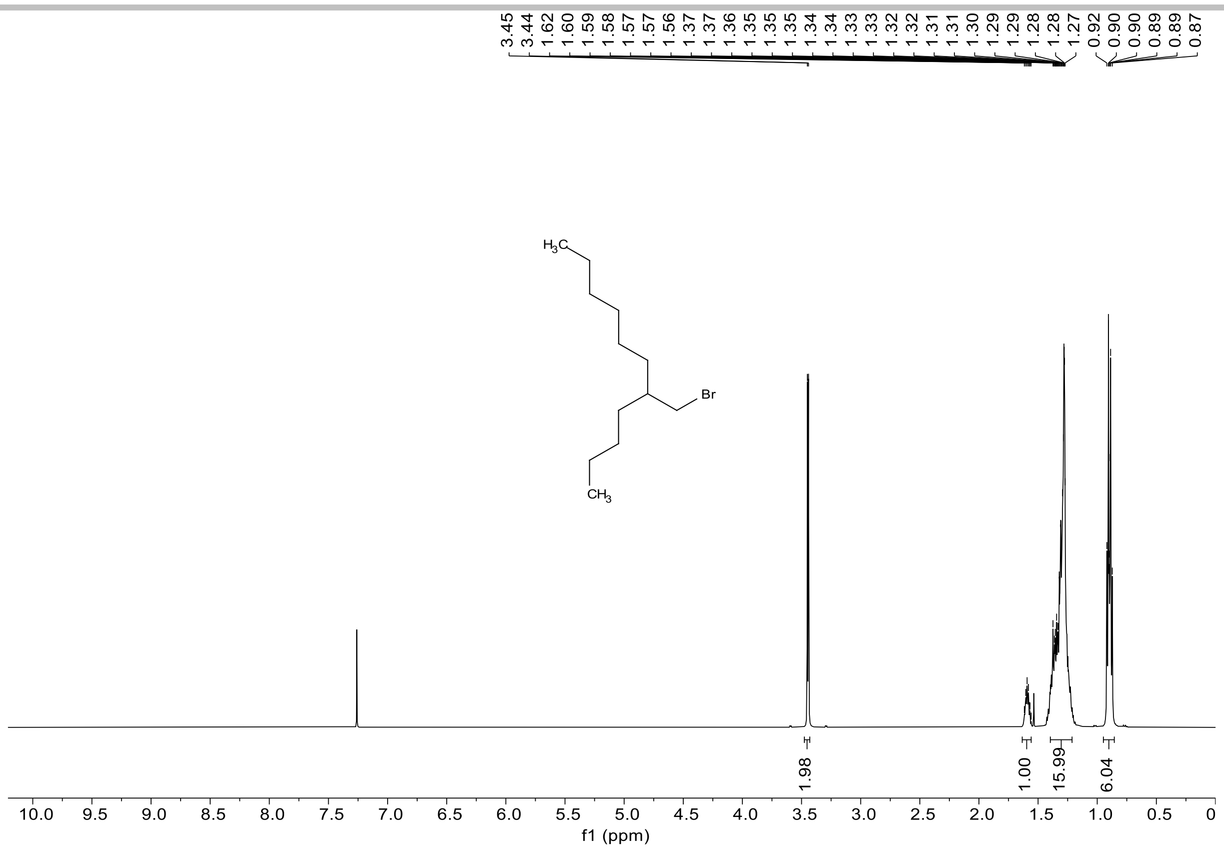

Supplementary Fig. 4 | ^{1}H NMR of **compound 6** (500 MHz, $CDCl_3$, 298 K).

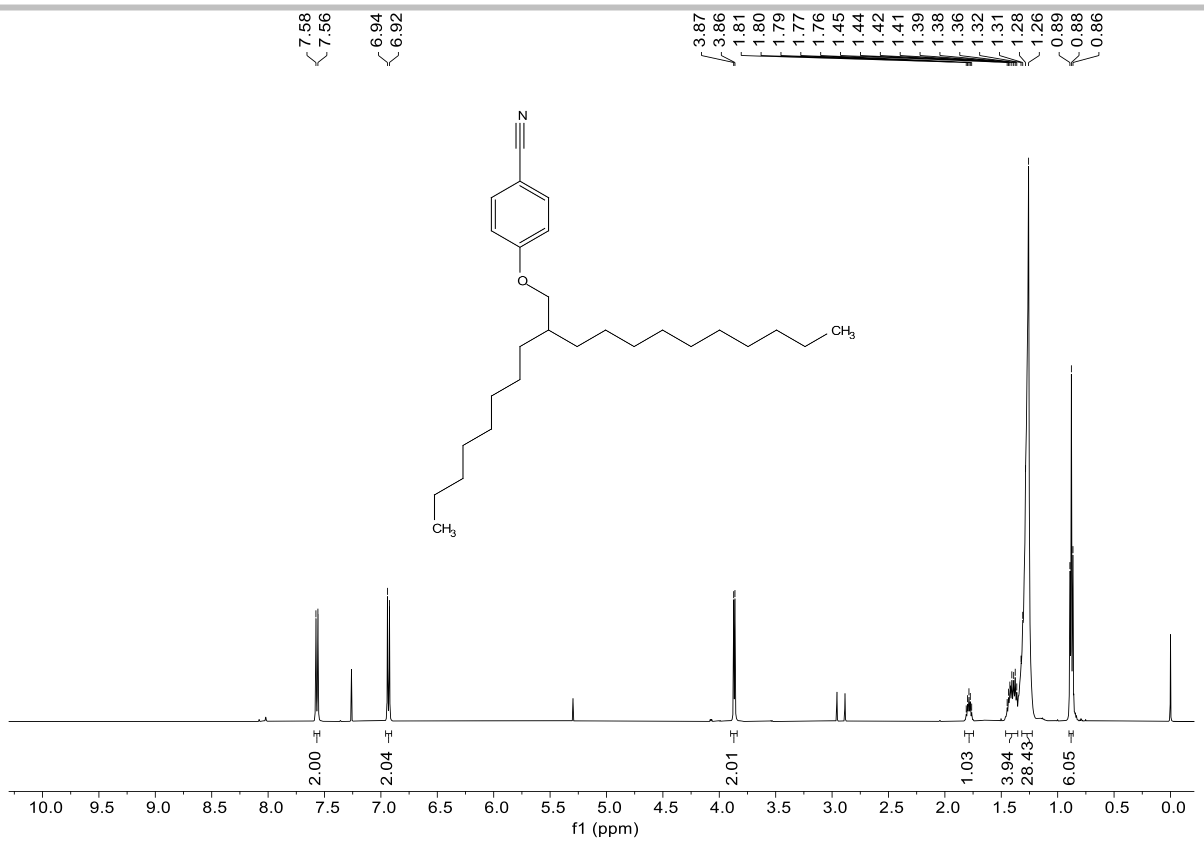

^{13}C NMR (101 MHz, $CDCl_3$) δ 162.71, 133.93, 119.35, 115.23, 108.34, 103.62, 71.31, 39.69, 39.54, 37.81, 36.12, 32.60, 31.94, 31.92, 31.27, 29.98, 29.81, 29.66, 29.62, 29.57, 29.49, 29.37, 29.33, 27.87, 26.82, 26.58, 22.70, 14.12.

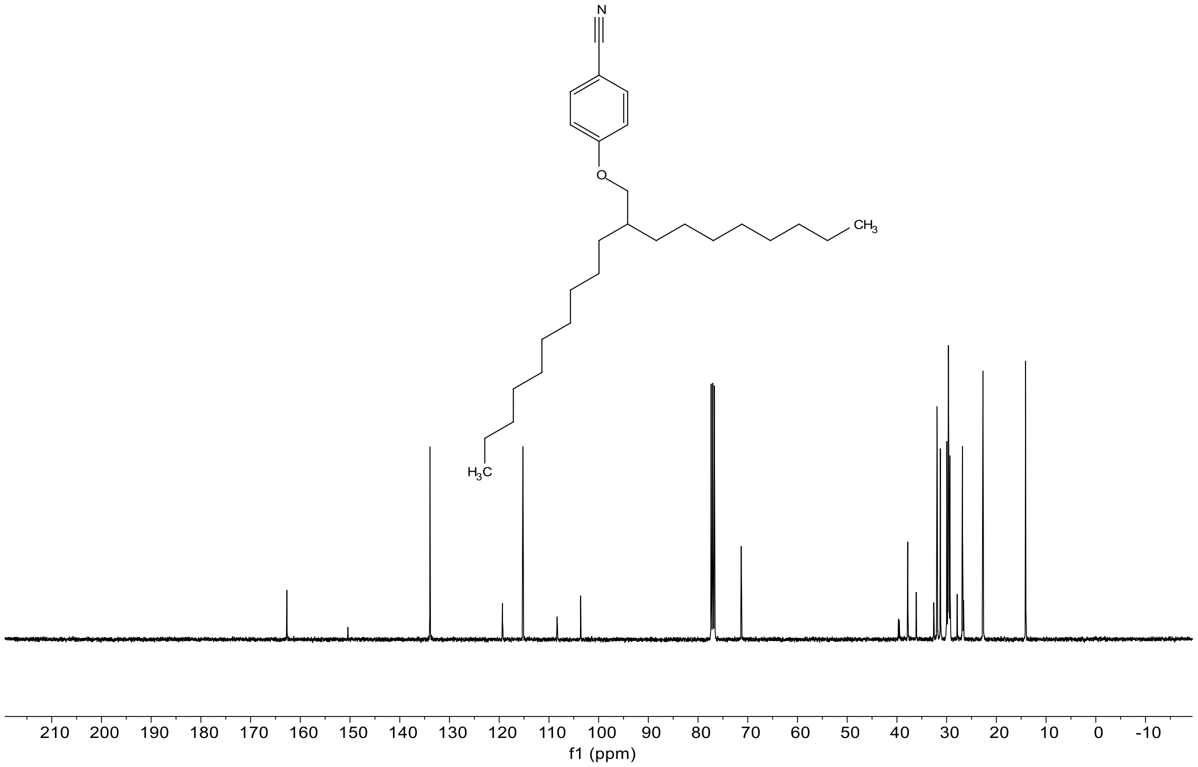

Supplementary Fig. 5 | ^{1}H NMR (500 MHz, 298 K) and ^{13}C NMR (101 MHz) of **compound 7** in $CDCl_3$.

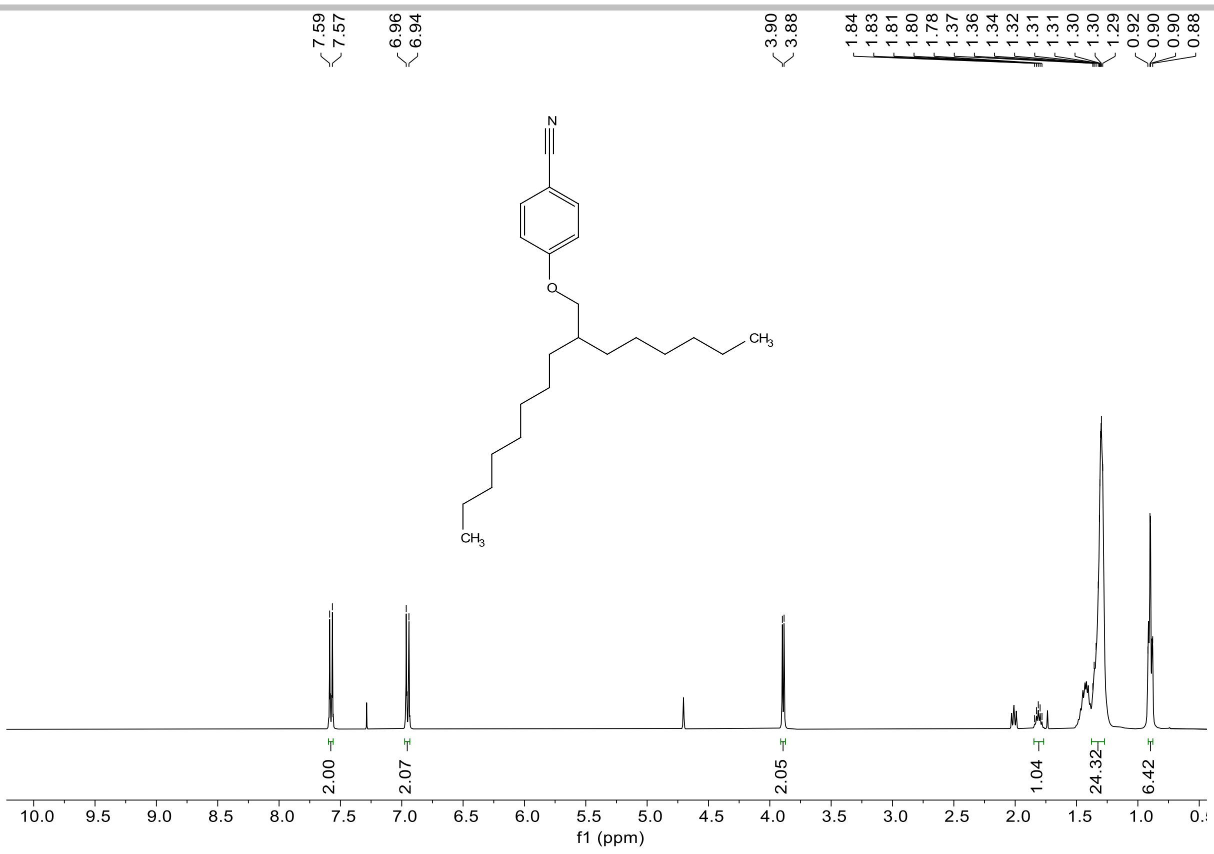

^{13}C NMR (101 MHz, $CDCl_3$) δ 162.70, 133.90, 119.31, 115.22, 108.35, 103.60, 71.29, 37.81, 36.10, 31.92, 31.90, 31.83, 31.28, 31.26, 29.97, 29.64, 29.56, 29.48, 29.14, 27.85, 26.81, 22.68, 14.10.

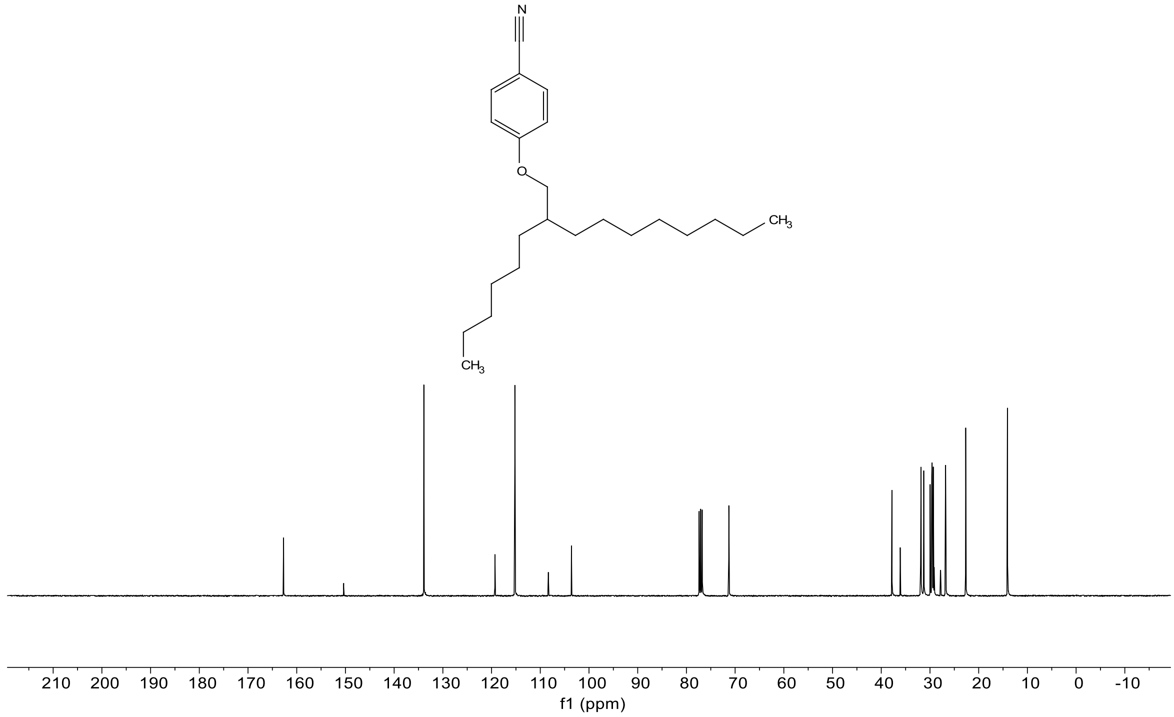

Supplementary Fig. 6 | ^{1}H NMR (500 MHz, 298 K) and ^{13}C NMR (101 MHz) of **compound 8** in $CDCl_3$.

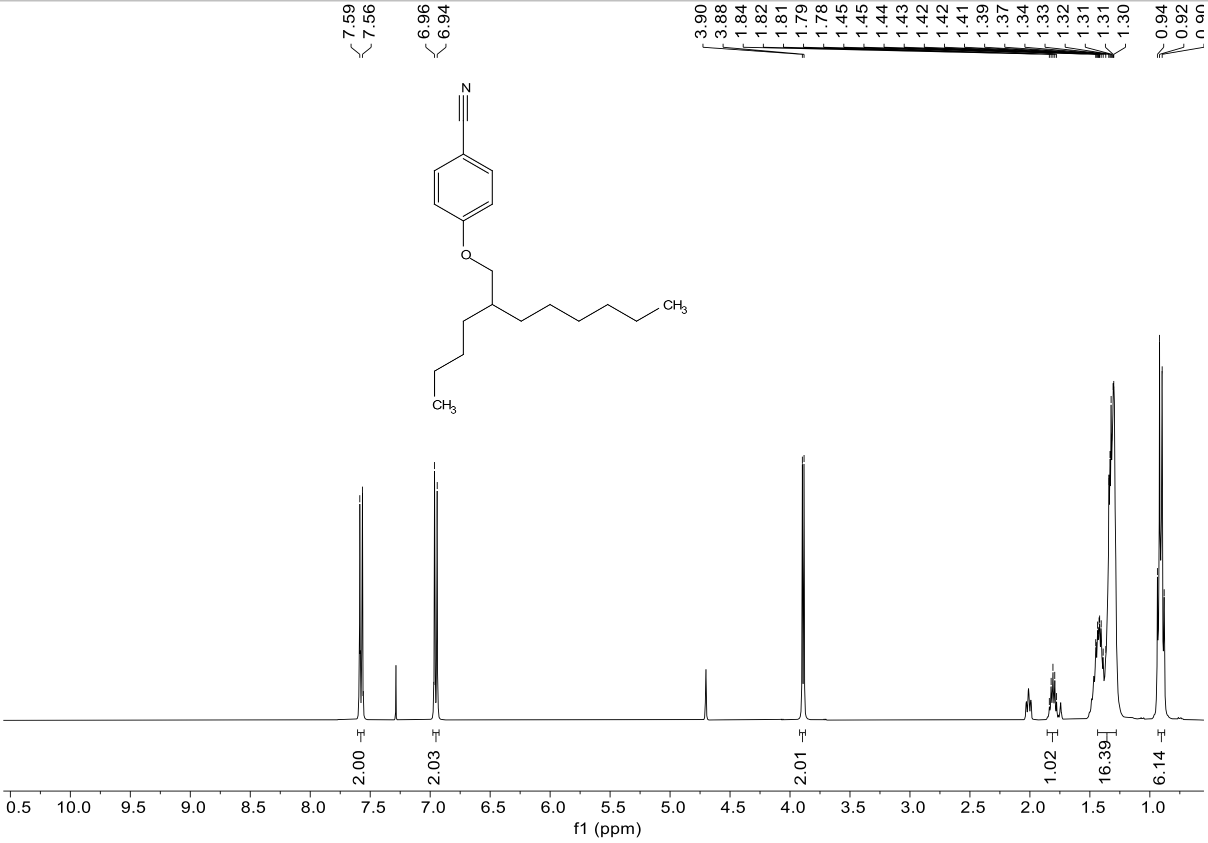

13C NMR (101 MHz, CDCl3) δ 162.70, 133.91, 119.32, 115.22, 108.34, 103.60, 71.29, 37.79, 31.82, 31.28, 30.96, 30.04, 29.63, 29.14, 29.03, 26.79, 23.01, 22.66, 14.09.

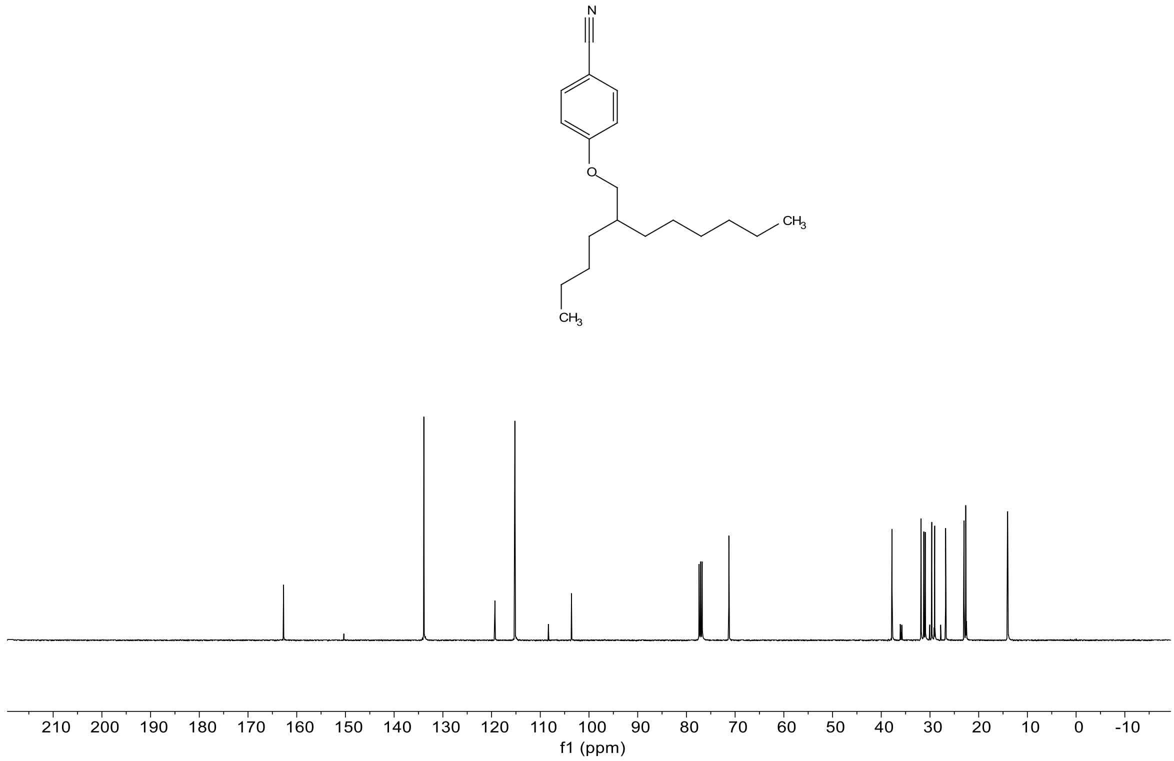

Supplementary Fig. 7 | ^{1}H NMR (500 MHz, 298 K) and ^{13}C NMR (101 MHz) of **compound 9** in $CDCl_3$.

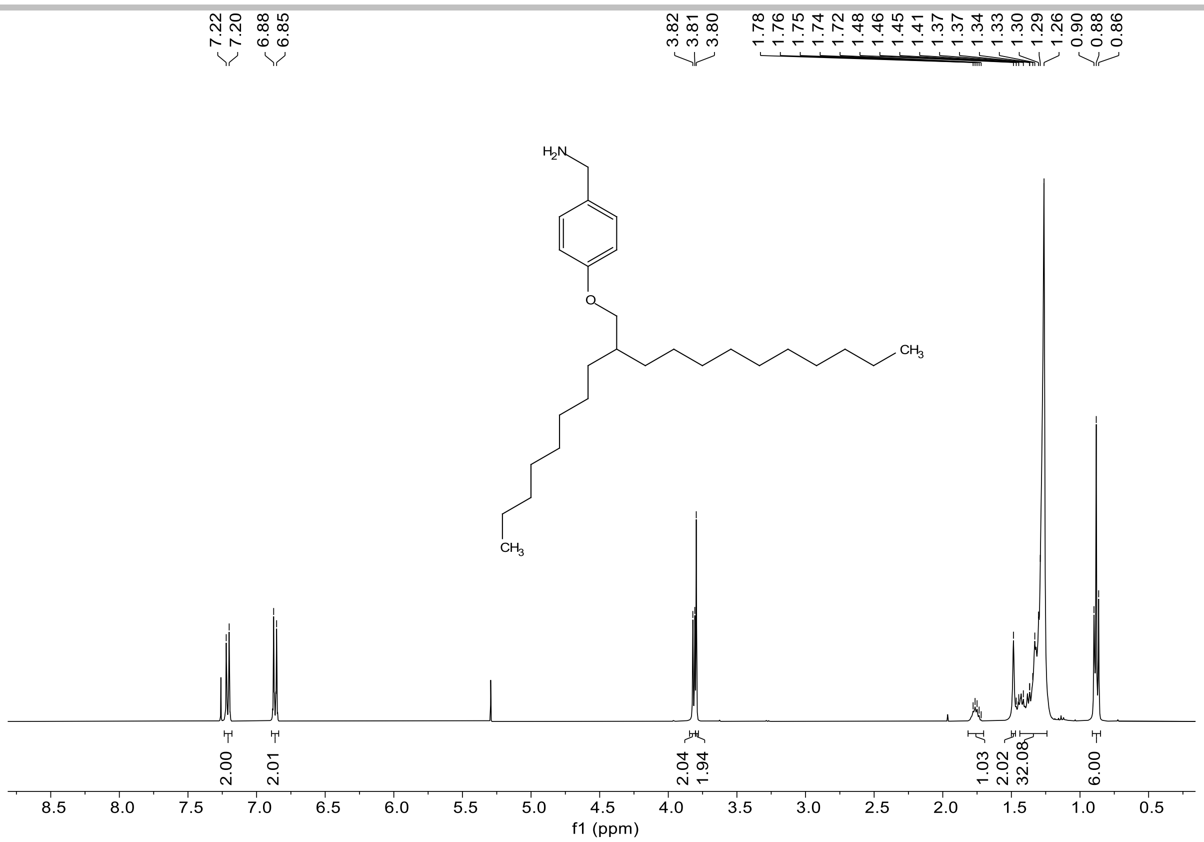

13C NMR (126 MHz, $CDCl_3$) δ 157.50, 127.36, 127.34, 113.60, 113.53, 113.49, 69.97, 44.56, 36.93, 36.44, 32.47, 30.90, 30.89, 30.34, 29.41, 29.01, 28.65, 28.62, 28.57, 28.33, 28.31, 27.95, 27.67, 25.81, 25.38, 21.67, 13.10.

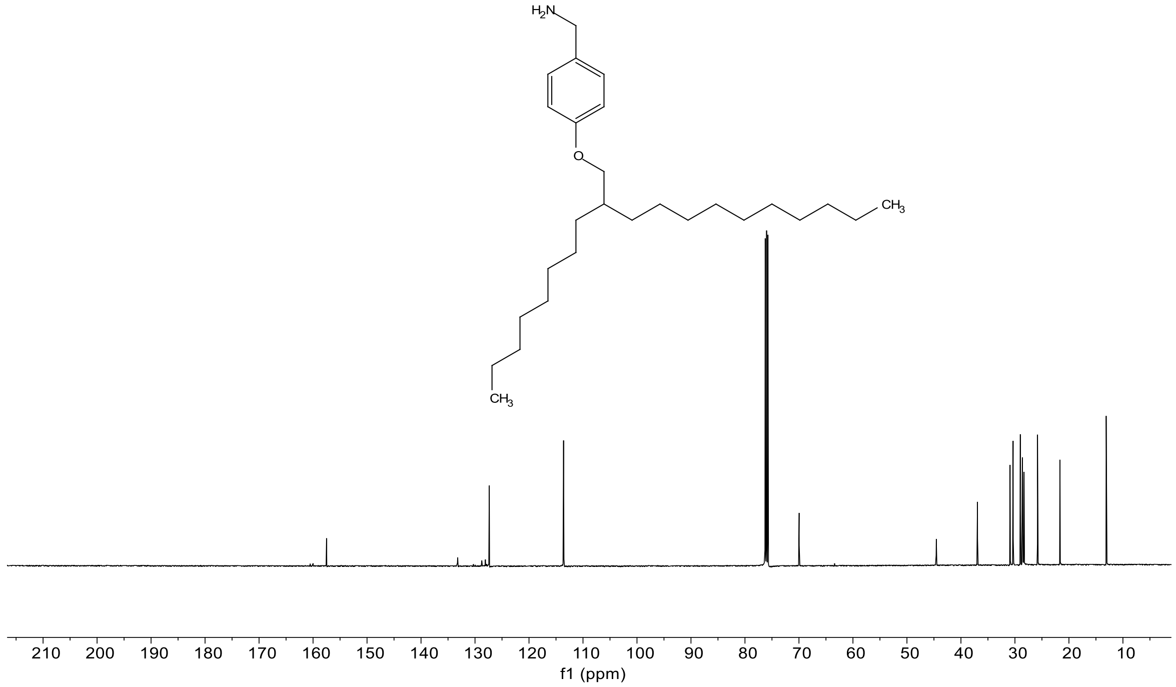

Supplementary Fig. 8 | ^{1}H NMR (500 MHz, 298 K) and ^{13}C NMR (126 MHz) of **compound 10** in $CDCl_3$.

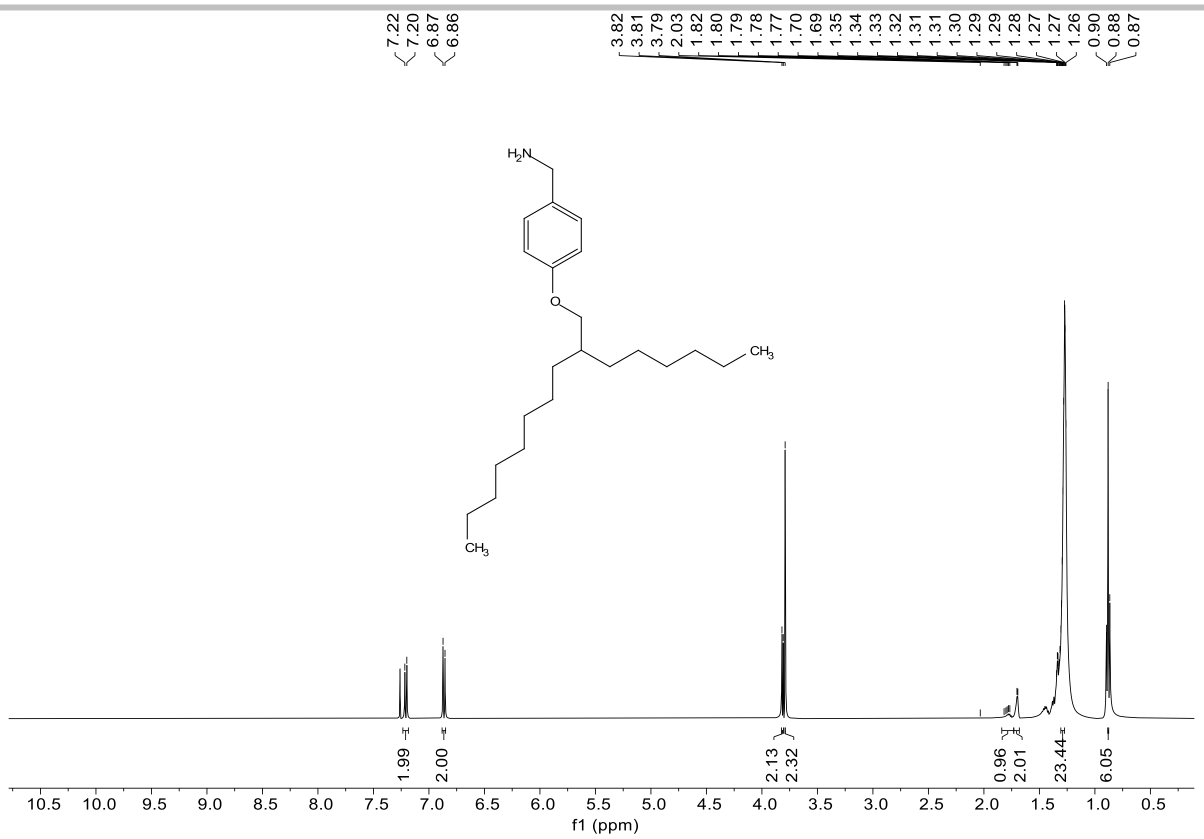

13C NMR (126 MHz, CDCl3) δ 157.47, 128.10, 127.78, 127.43, 115.35, 113.51, 69.98, 44.47, 36.94, 30.89, 30.84, 30.35, 30.34, 29.01, 28.99, 28.68, 28.66, 28.57, 28.31, 25.81, 25.79, 21.66, 13.09.

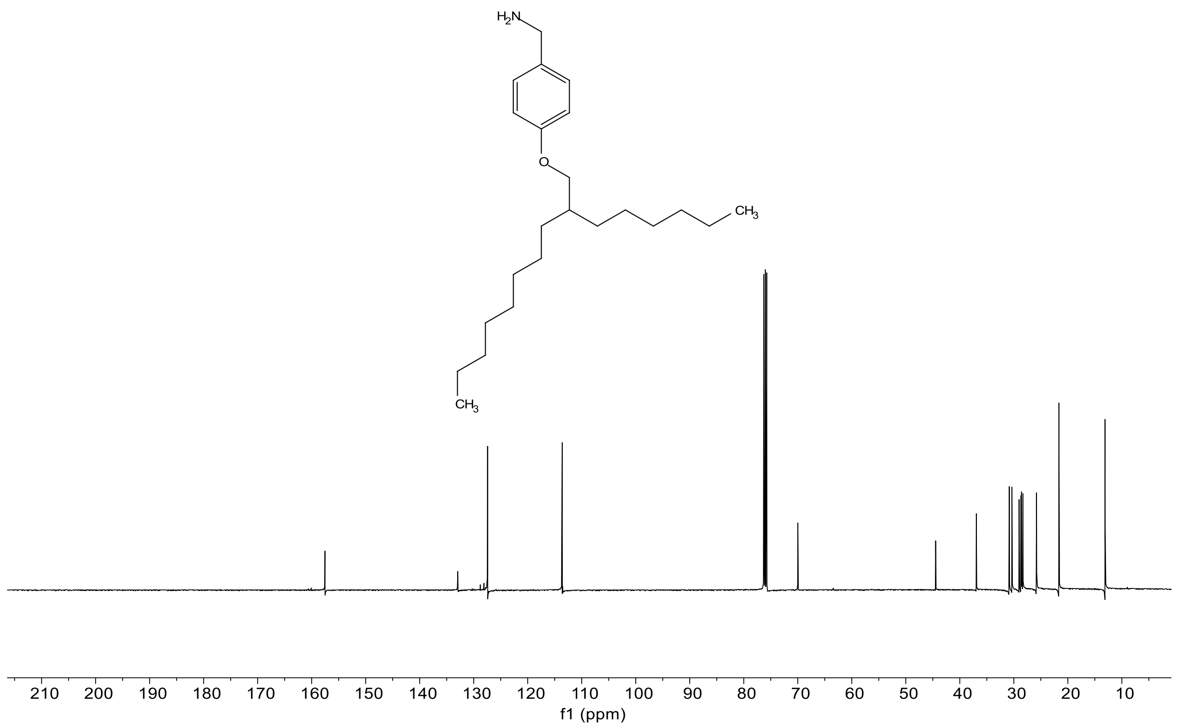

Supplementary Fig. 9 | ^{1}H NMR (500 MHz, 298 K) and ^{13}C NMR (126 MHz) of **compound 11** in $CDCl_3$.

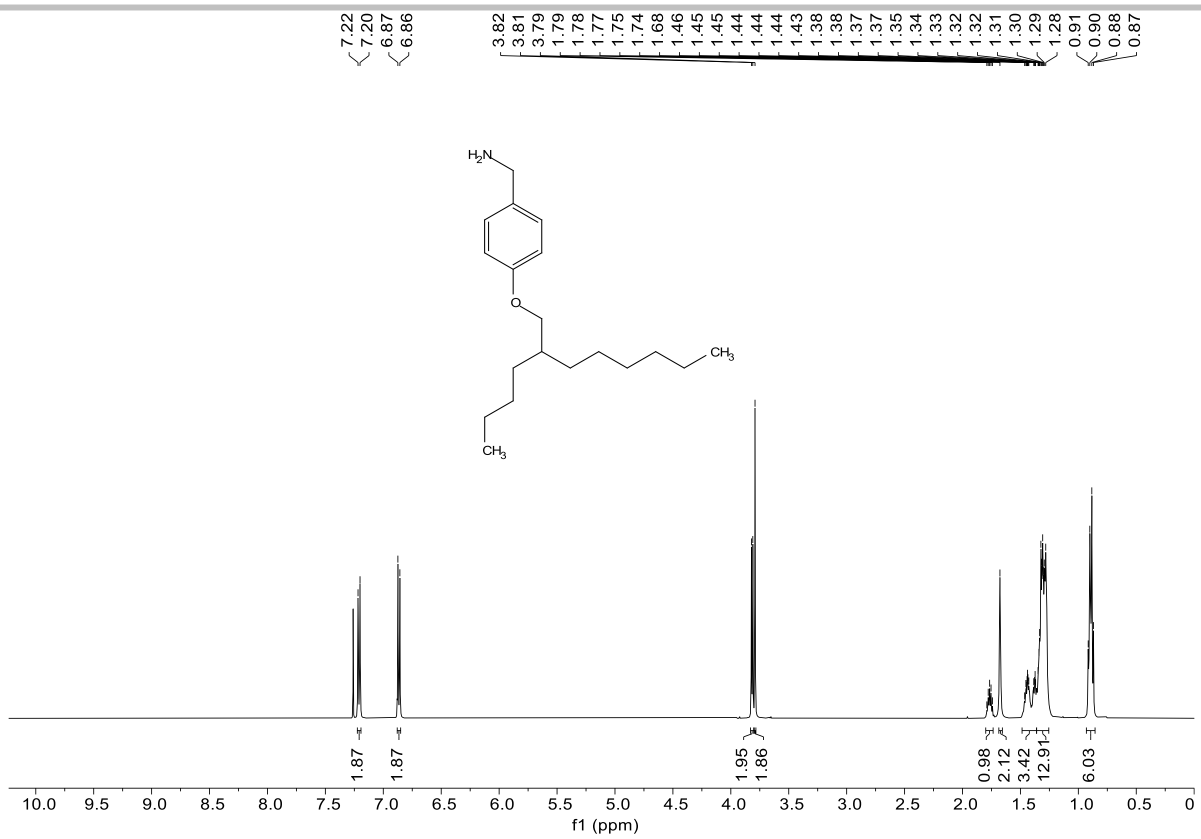

^{13}C NMR (126 MHz, $CDCl_3$) δ 157.47, 127.85, 127.34, 113.59, 113.49, 113.18, 69.95, 44.61, 36.92, 30.84, 30.36, 30.04, 28.67, 28.34, 28.03, 25.79, 22.04, 21.65, 13.09.

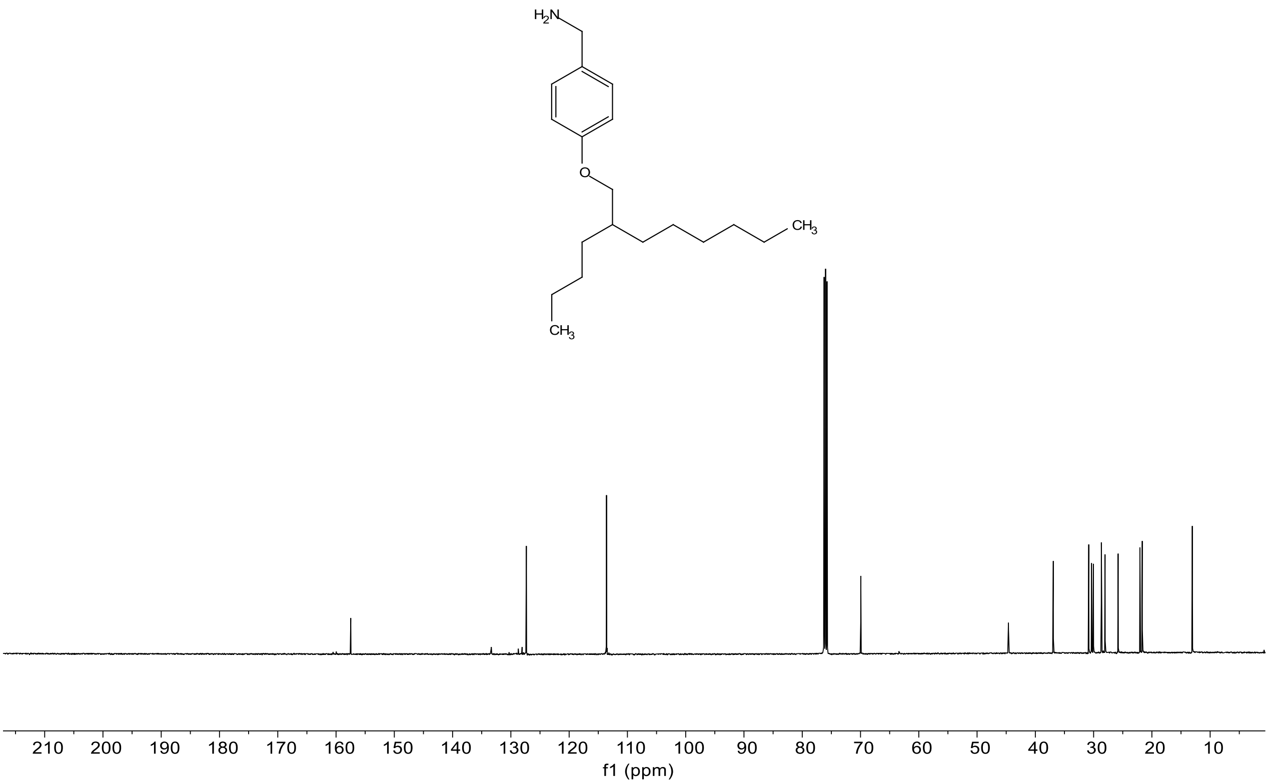

Supplementary Fig. 10 | ^{1}H NMR (500 MHz, 298 K) and ^{13}C NMR (126 MHz) of **compound 12** in $CDCl_3$.

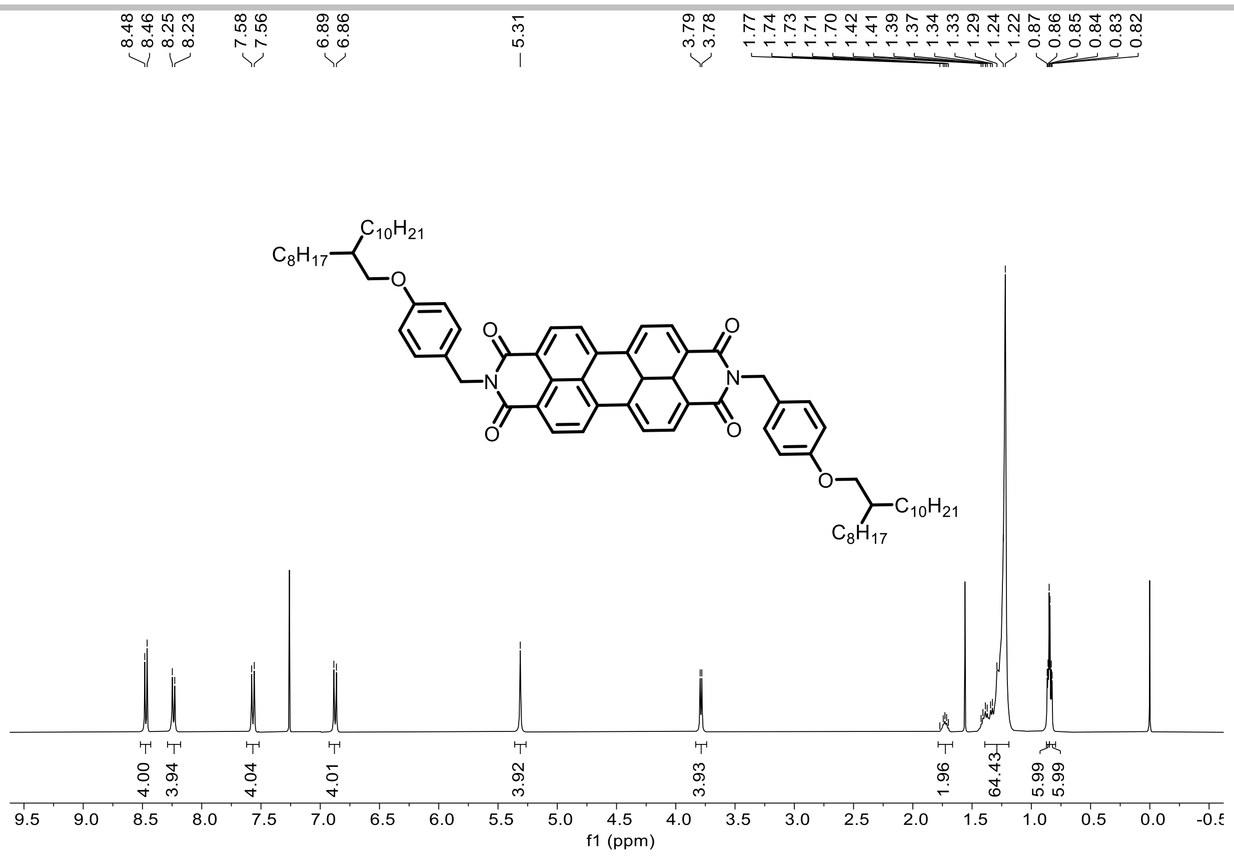

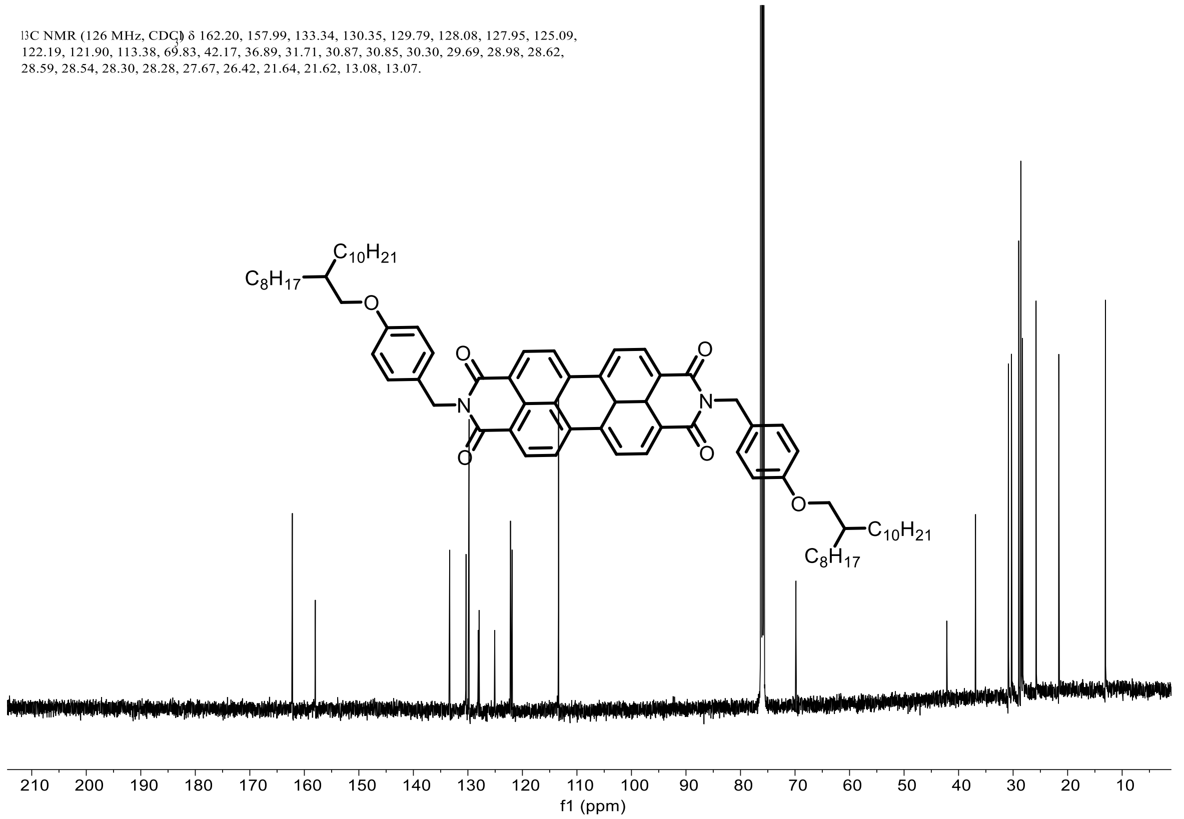

Supplementary Fig. 11 | ^{1}H NMR (500 MHz, 298 K) and ^{13}C NMR (126 MHz) of **8,10-ab-PBI** in $CDCl_3$.

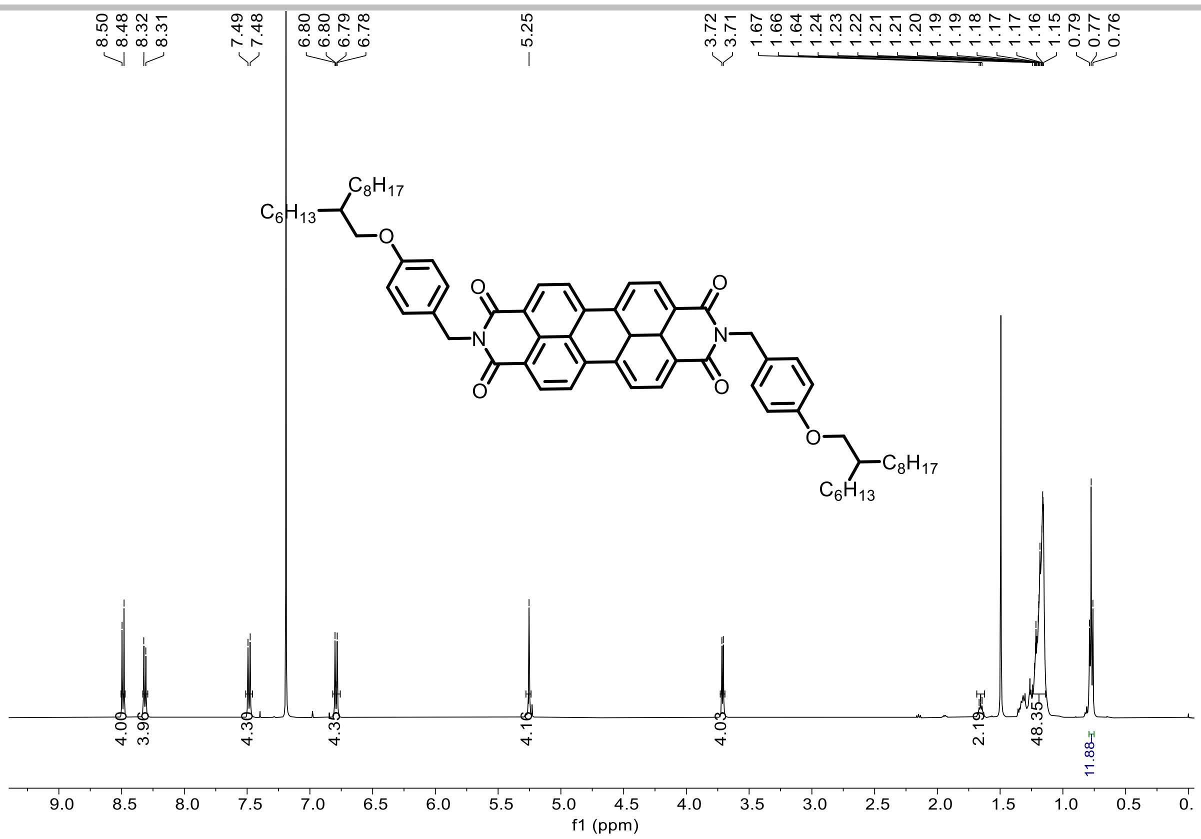

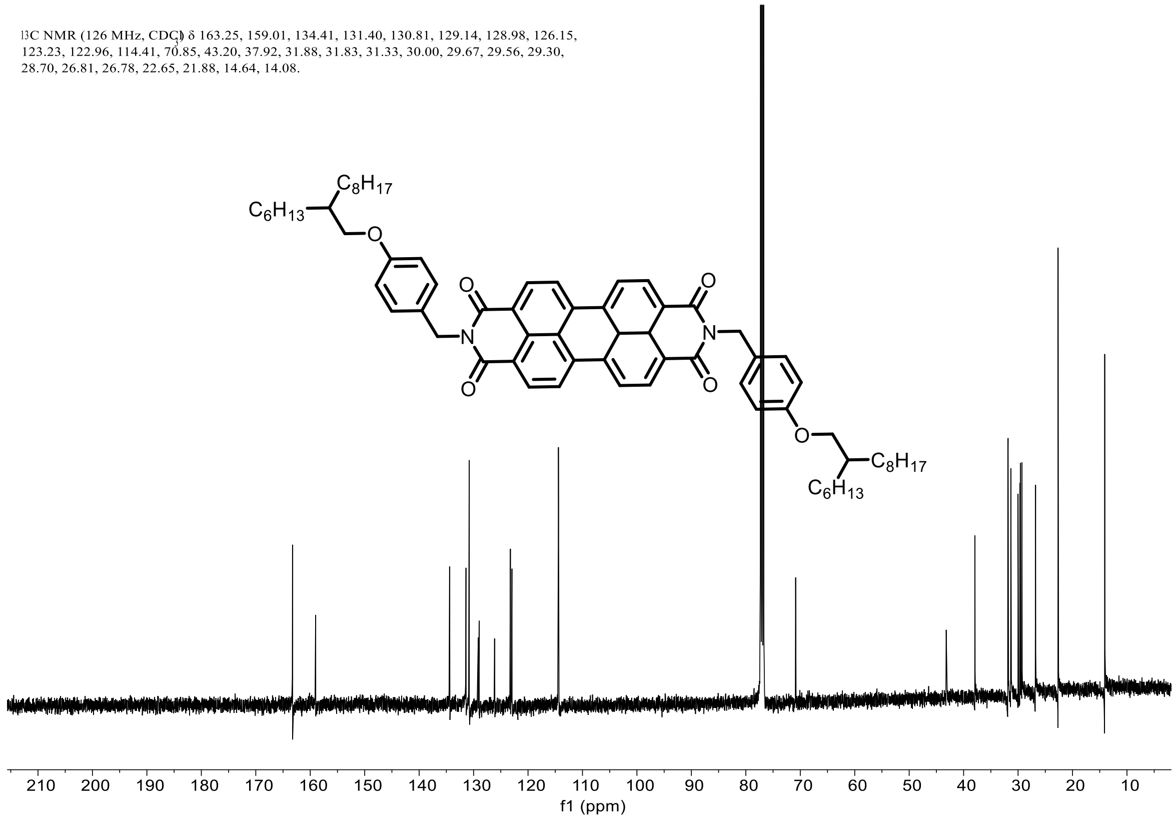

Supplementary Fig. 12 | ^{1}H NMR (500 MHz, 298 K) and ^{13}C NMR (126 MHz) of **6,8-ab-PBI** in $CDCl_3$.

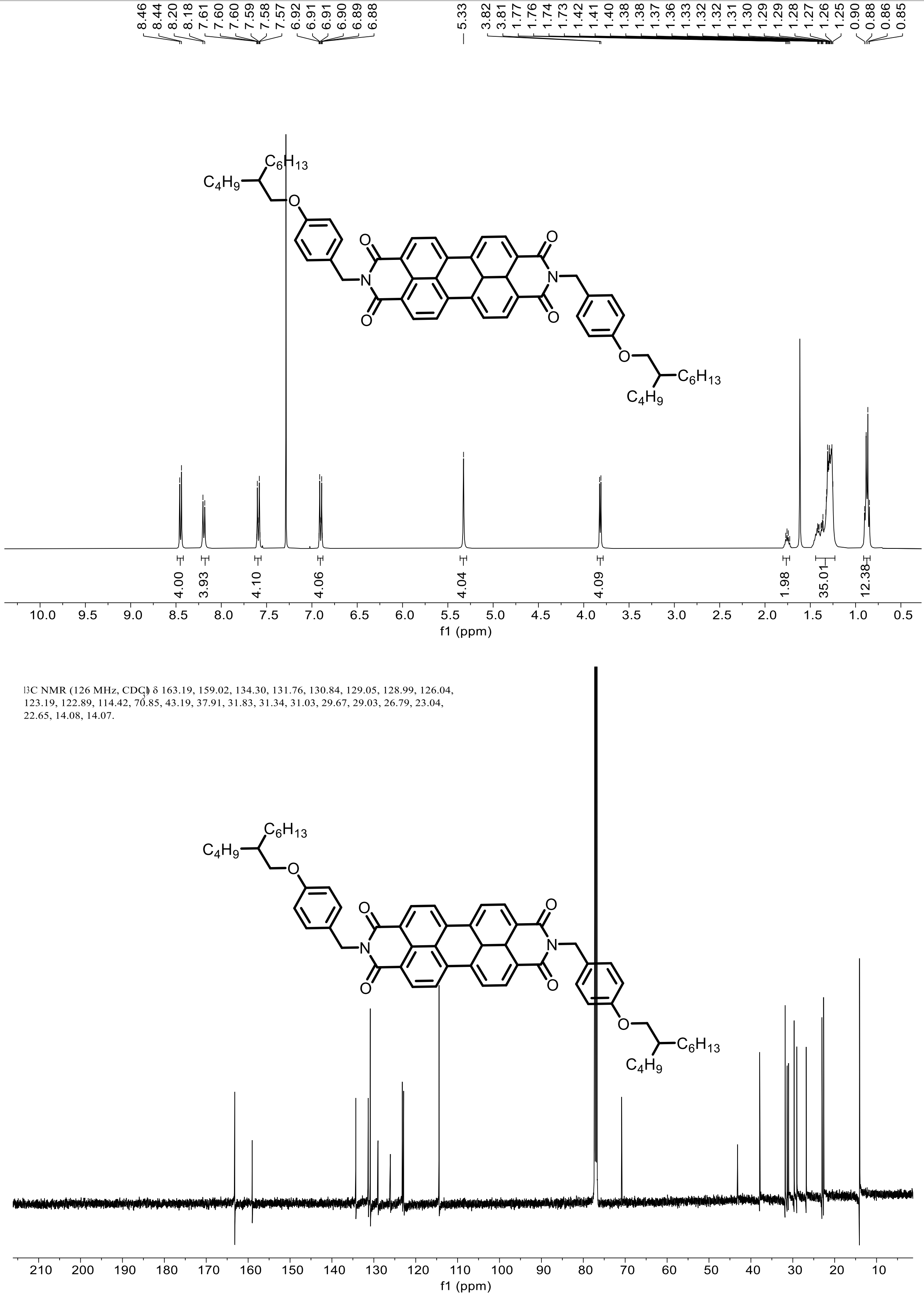

Supplementary Fig. 13 | ^{1}H NMR (500 MHz, 298 K) and ^{13}C NMR (126 MHz) of **4,6-ab-PBI** in $CDCl_3$.

4. Single Crystal X-ray Analysis

The crystals were kept at 150 K / 300 K during data collection. Using Olex2, the structure was solved with the ShelXT structure solution program by intrinsic Phasing, and refined with the ShelXL refinement package by least squares minimization. [1,2,3] The crystal data and structure refinement in detail were summarized in Supplementary Table 1 and Supplementary Table 2. The large R_{int} was owing to the soft nature of single crystal originated from weak interactions between lamellar structures. It's a pity that we didn't get the crystal data of **8,10-ab-PBI** at 300K.

Supplementary Table 1 | Crystal data at 150K and structure refinement for **4,6-ab-PBI**, **6,8-ab-PBI** and **8,10-ab-PBI**.

PBIs	4,6-ab-PBI	6,8-ab-PBI	8,10-ab-PBI
**CCDC number**	2211699	2211700	1974107
**Empirical formula**	$C_{62}H_{70}N_2O_6$	$C_{70}H_{86}N_2O_6$	$C_{78}H_{102}N_2O_6$
**Formula weight**	939.20	1051.40	1163.61
**Temperature/K**	149.98(10)	149.99(10)	119.99(18)
**Crystal system**	monoclinic	monoclinic	monoclinic
**Space group**	$P2_1$/c	$P2_1$/c	$I2$/a
***a*/Å**	29.666(6)	34.166(9)	17.781(3)
***b*/Å**	4.7981(14)	4.8536(12)	4.8015(9)
***c*/Å**	18.069(3)	17.799(4)	77.112(17)
**α/°**	90	90	90
**β/°**	100.42(2)	94.14(2)	93.894(15)
**γ/°**	90	90	90
**Volume/Å^3**	2529.5(10)	2943.9(12)	6568(2)
Z	2	2	4
**ρ_{calc}g/cm^3**	1.233	1.186	1.177
**μ/mm^{-1}**	0.616	0.579	0.563
***F* (000)**	1008.0	1136.0	2528.0
**Crystal size/mm^3**	0.12 × 0.04 × 0.02	0.1 × 0.04 × 0.02	0.09 × 0.08 × 0.04
**Radiation**	CuKα (λ = 1.54184)	CuKα (λ = 1.54184)	CuKα (λ = 1.54184)
**2Θ range for data collection/°**	9.092 to 134.096	5.186 to 134.142	6.894 to 148.416
**Index ranges**	$-23 \le h \le 35$, $-5 \le k \le 5$, $-21 \le l \le 21$	$-40 \le h \le 32$, $-5 \le k \le 4$, $-13 \le l \le 21$	$-20 \le h \le 21$, $-4 \le k \le 5$, $-96 \le l \le 95$
**Reflections collected**	10858	16924	24896
**Independent reflections**	4369 [R_{int} = 0.1664, R_{sigma} = 0.2180]	5149 [R_{int} = 0.1049, R_{sigma} = 0.0677]	6302 [R_{int} = 0.1357, R_{sigma} = 0.1760]
**Data/restraints/parameters**	4369/6/328	5149/101/354	6302/39/390
**Goodness-of-fit on *F*^{2}**	1.094	1.276	1.133
**Final *R* indexes [I>=2σ (I)]**	R_1 = 0.1738, wR_2 = 0.4001	R_1 = 0.2382, wR_2 = 0.6414	R_1 = 0.1743, wR_2 = 0.4153
**Final *R* indexes [all data]**	R_1 = 0.3037, wR_2 = 0.4780	R_1 = 0.2977, wR_2 = 0.6942	R_1 = 0.2695, wR_2 = 0.4976

Supplementary Table 2 | Crystal data at 300K and structure refinement for **4,6-ab-PBI** and **6,8-ab-PBI**.

PBIs	4,6-ab-PBI	6,8-ab-PBI
**CCDC number**	2211720	2211721
**Empirical formula**	$C_{62}H_{70}N_2O_6$ (4,6-ab-PBI)	$C_{70}H_{86}N_2O_6$ (6,8-ab-PBI)
**Formula weight**	939.20	1051.40
**Temperature/K**	282(10)	300.9(6)
**Crystal system**	monoclinic	monoclinic
**Space group**	$P2_1$/c	*C*c
a/Å	30.426(14)	68.562(13)
b/Å	4.8866(17)	4.9786(7)
c/Å	18.124(7)	18.0025(16)
α/°	90	90
β/°	106.40(4)	95.894(12)
γ/°	90	90
**Volume/Å^3**	2585.0(18)	6112.6(16)
Z	2	4
ρ_{calc}**g/cm^3**	1.207	1.142
μ**/mm^{-1}**	0.603	0.557
F **(000)**	1008.0	2272.0
**Crystal size/mm^3**	0.16 × 0.05 × 0.03	0.11 × 0.06 × 0.04
**Radiation**	CuKα (λ = 1.54184)	CuKα (λ = 1.54184)
**2Θ range for data collection/°**	6.056 to 134.158	7.778 to 134.144
**Index ranges**	$-36 \le h \le 32$, $-5 \le k \le 3$, $-21 \le l \le 21$	$-80 \le h \le 80$, $-5 \le k \le 3$, $-21 \le l \le 21$
**Reflections collected**	17519	25531
**Independent reflections**	4584 [R_{int} = 0.1788, R_{sigma} = 0.1880]	10082 [R_{int} = 0.0675, R_{sigma} = 0.1017]
**Data/restraints/parameters**	4584/78/316	10082/289/659
**Goodness-of-fit on** F^2	1.054	1.259
**Final** ***R*** **indexes [I>=2σ (I)]**	R_1 = 0.1777, wR_2 = 0.3962	R_1 = 0.1454, wR_2 = 0.3759
**Final** ***R*** **indexes [all data]**	R_1 = 0.3594, wR_2 = 0.5120	R_1 = 0.2145, wR_2 = 0.4430

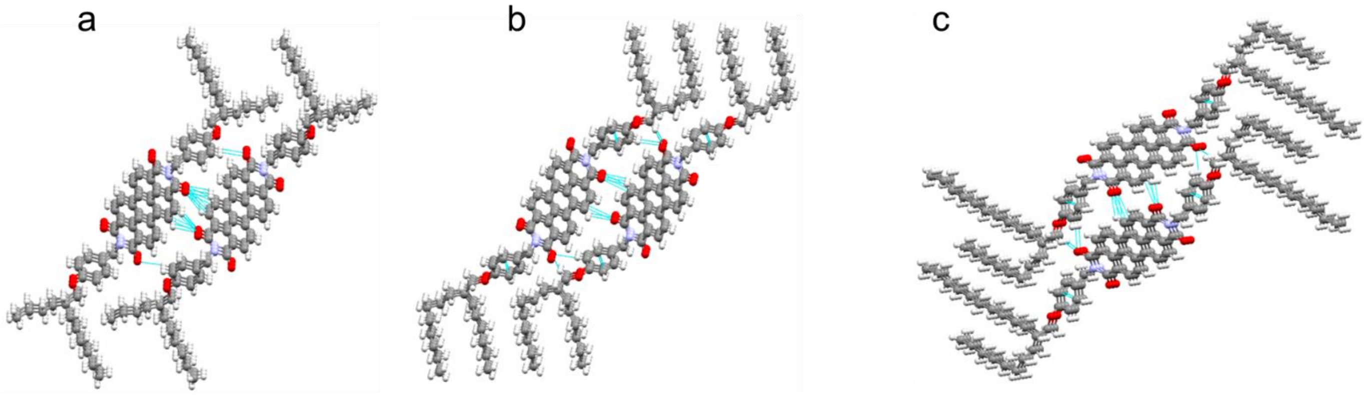

Supplementary Fig. 14 | The multiple intermolecular C-H…O interaction of **a 4,6-ab-PBI**, **b 6,8-ab-PBI** and **c 8,10-ab-PBI** formed between adjacent molecular columns.

5. Photophysical Properties of Monomers

Supplementary Table 3 | Calculated CT coupling (J_{CT}) and Coulombic coupling (J_{Coul}) of PBIs. The molecular geometries of monomers and dimers were optimized by Gaussian program at the DFT level using the B3LYP functional[4,5] and the 6-31G** basis set.[6,7] The short-range exciton coupling energy (J_{CT}) was calculated with $J_{CT} = -2t_e t_h / (E_{CT} - E_{S1})$ [8,9,10] (E_{CT}-E_{S1} = 200 meV), where t_e and t_h are the hole- and electron- transfer integrals respectively, E_{CT} is the energy of the CT exciton, and E_{S1} is the energy of the local Frenkel exciton state. The values for CT integrals t_e and t_h of dimers, depending on the wavefunction overlap of LUMO-LUMO and HOMO-HOMO orbital of the nearest-neighbor PBIs molecules, were calculated using the polarization-including energy splitting in the dimer model.[11,12] Single point were calculated using m062x functional and 6-311G** basis set. Long-range coupling energy (J_{Coul}), was evaluated using electrostatic potential (TrEsp) method.[13] Where the time-dependent DFT was calculated using m062x functional and TZVP basis set. And the vacuum dielectric constant ε was chosen to be 3 when considering the environmental impact in the solid state.[14]

PBI	J_{coul}/ε [meV]	t_h [meV]	t_e [meV]	J_{CT} [meV]
**4,6-ab-PBI**	38.52	117.11	23.01	-26.94
**6,8-ab-PBI**	31.82	92.02	38.93	-35.82
**8,10-ab-PBI**	37.00	-101.75	-44.97	-45.75

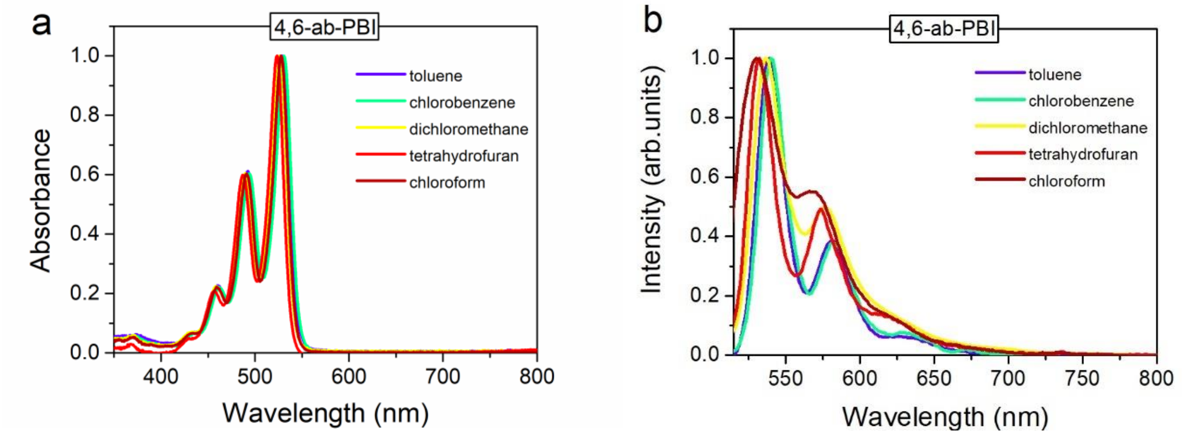

Supplementary Fig. 15 | a UV/Vis absorption (1×10^{-5} M) and **b** fluorescence emission (1×10^{-7} M, λ_{ex} = 490 nm) spectra of **4,6-ab-PBI** in solvents of varying polarity.

Supplementary Table 4 | Absorption and fluorescence properties of **4,6-ab-PBI** in solvents of varying polarity.

Solvent	λ_{abs} [a]/nm	ε_{max}/M^{-1} cm^{-1}	λ_{em} [a]/nm	$\Delta\tilde{\nu}_{Stokes}$/ cm^{-1}	τ/ ns	Φ_F/%
toluene	529	75200	539	351	1.6 ± 0.01	25
chlorobenzene	531	71100	540	314	0.6 ± 0.01 (79%), 5.5 ± 0.61 (21%)	10
dichloromethane	527	58400	537	353	0.3 ± 0.01 (42%), 4.6 ± 0.21 (58%)	1
tetrahydrofuran	523	72400	532	323	0.2 ± 0.01 (75%), 4.7 ± 0.45 (25%)	2
chloroform	528	88100	530	71	0.3 ± 0.01 (40%), 4.1 ± 0.17 (60%)	2

[a]The given values are those for the maximum of the largest wavelength absorption (1×10^{-5} M) and emission (1×10^{-7} M) band.

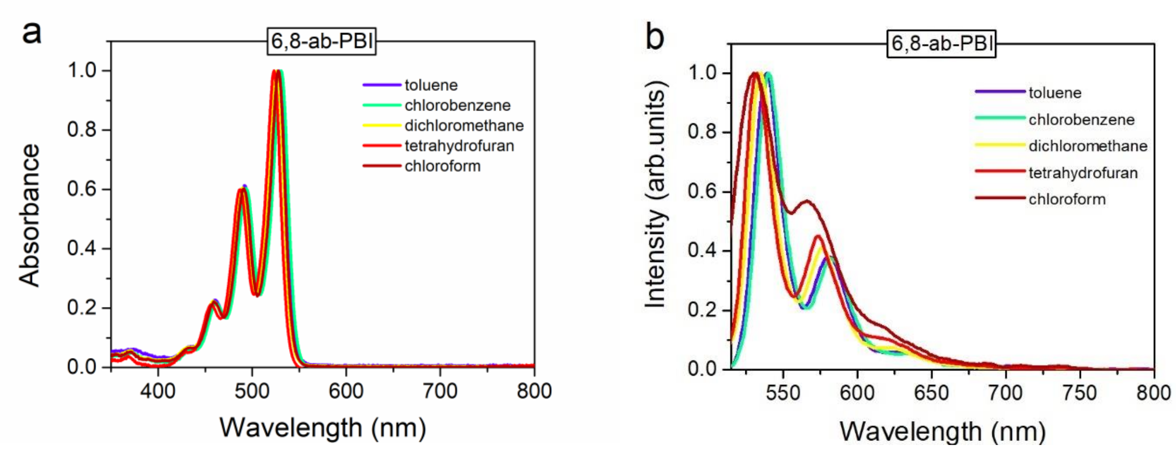

Supplementary Fig. 16 | a UV/Vis absorption (1×10^{-5} M) and **b** fluorescence emission (1×10^{-7} M, λ_{ex} = 490 nm) spectra of **6,8-ab-PBI** in solvents of varying polarity.

Supplementary Table 5 | Absorption and fluorescence properties of **6,8-ab-PBI** in solvents of varying polarity.

Solvent	λ_{abs} ^{a}/nm	ε_{max}/M^{-1} cm^{-1}	λ_{em}^{a}/nm	$\Delta\tilde{\nu}_{Stokes}$ / cm^{-1}	τ/ ns	Φ_F/%
toluene	529	71300	539	351	1.6 ± 0.02	22
chlorobenzene	531	82100	540	314	0.7 ± 0.01 (81%), 6.6 ± 0.90 (19%)	9
dichloromethane	526	73600	534	285	0.3 ± 0.01 (46%), 5.0 ± 0.27 (54%)	1
tetrahydrofuran	523	77900	532	323	0.4 ± 0.01 (68%), 4.2 ± 0.32 (32%)	1
chloroform	528	88000	530	71	0.4 ± 0.01 (72%), 4.7 ± 0.47 (28%)	2

[a]The given values are those for the maximum of the largest wavelength absorption (1×10^{-5} M) and emission (1×10^{-7} M) band.

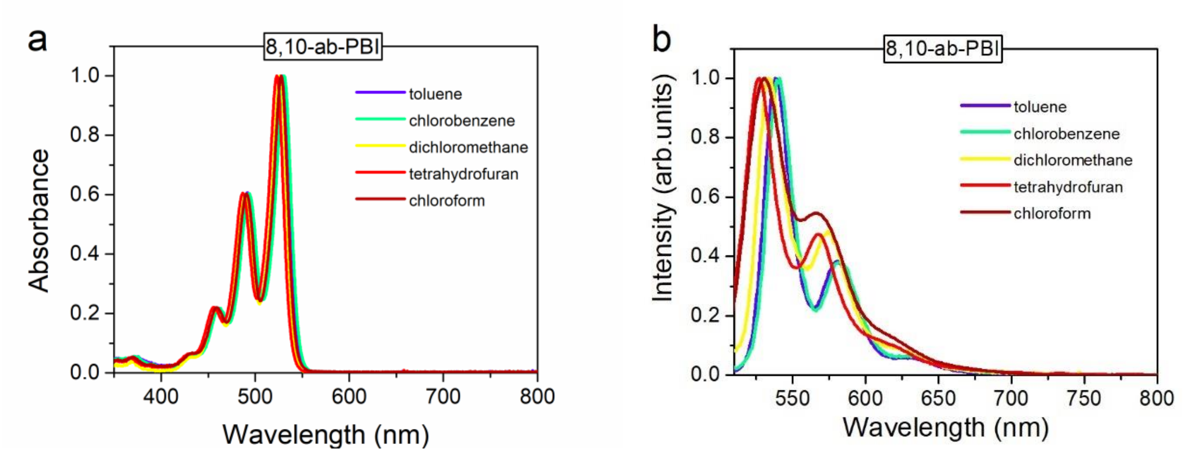

Supplementary Fig. 17 | a UV/Vis absorption (1×10^{-5} M) and **b** fluorescence emission (1×10^{-7} M, λ_{ex} = 490 nm) spectra of **8,10-ab-PBI** in solvents of varying polarity.

Supplementary Table 6 | Absorption and fluorescence properties of **8,10-ab-PBI** in solvents of varying polarity.

Solvent	λ_{abs} [a]/nm	ε_{max}/M^{-1} cm^{-1}	λ_{em} [a]/nm	$\Delta\tilde{\nu}_{Stokes}$ / cm^{-1}	τ/ ns	Φ_F/%
toluene	529	52700	539	351	1.6 ± 0.01	23
chlorobenzene	531	69400	540	314	0.6 ± 0.01 (72%),<br>5.1 ± 0.41 (28%)	7
dichloromethane	526	61300	534	285	0.3 ± 0.01 (55%),<br>4.9 ± 0.28 (45%)	2
tetrahydrofuran	523	66200	532	323	0.3 ± 0.01 (60%),<br>4.7 ± 0.41 (40%)	2
chloroform	527	69000	530	107	0.3 ± 0.01 (52%),<br>4.3 ± 0.27 (48%)	1

[a]The given values are those for the maximum of the largest wavelength absorption (1×10^{-5} M) and emission (1×10^{-7} M) band.

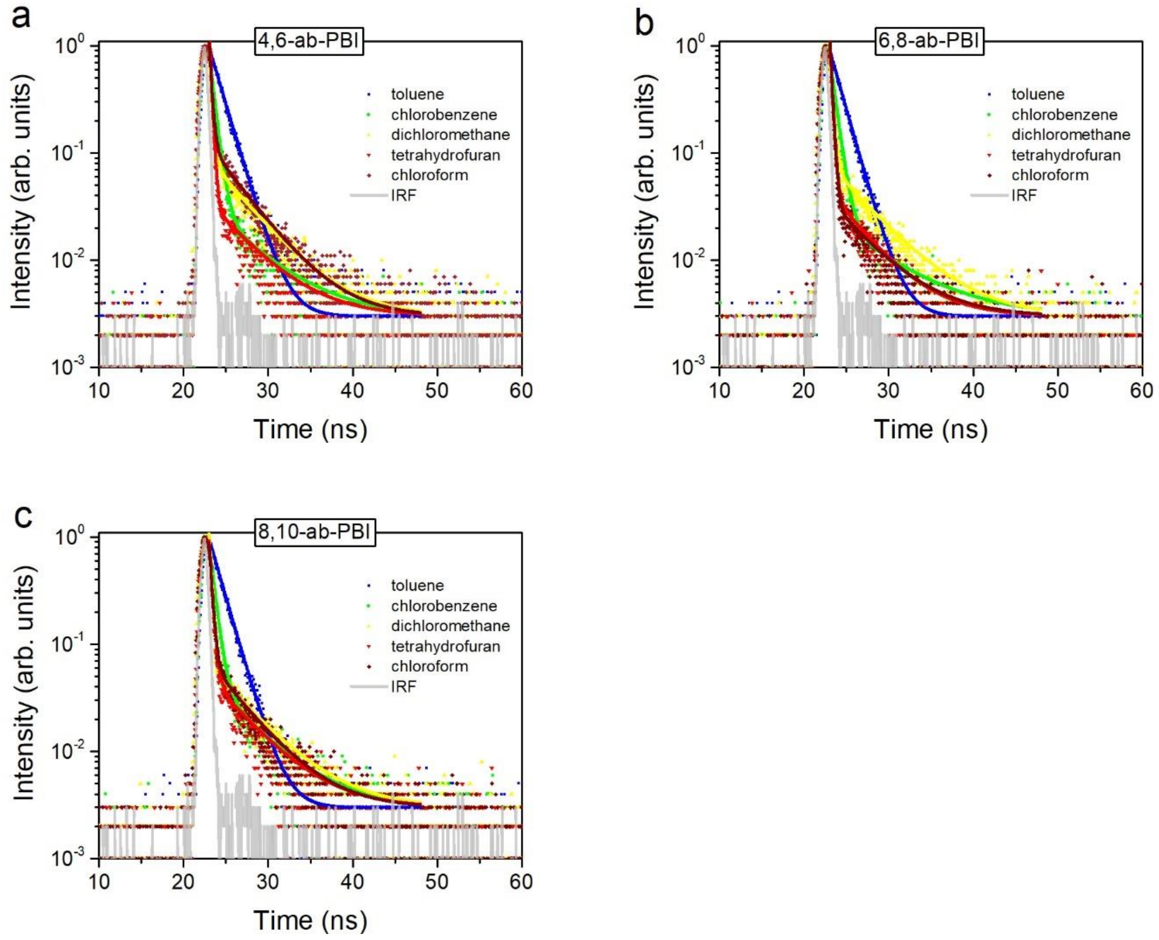

Supplementary Fig. 18 | Fluorescent decay traces of **a 4,6-ab-PBI**, **b 6,8-ab-PBI** and **c 8,10-ab-PBI** in solvents of varying polarity; the instrumental response function (IRF; in grey) is given for comparison. Solid lines are bi-exponential fits to the experimental data, obtained by deconvolution with the IRF; results of the fitting are given in Supplementary Table 6.

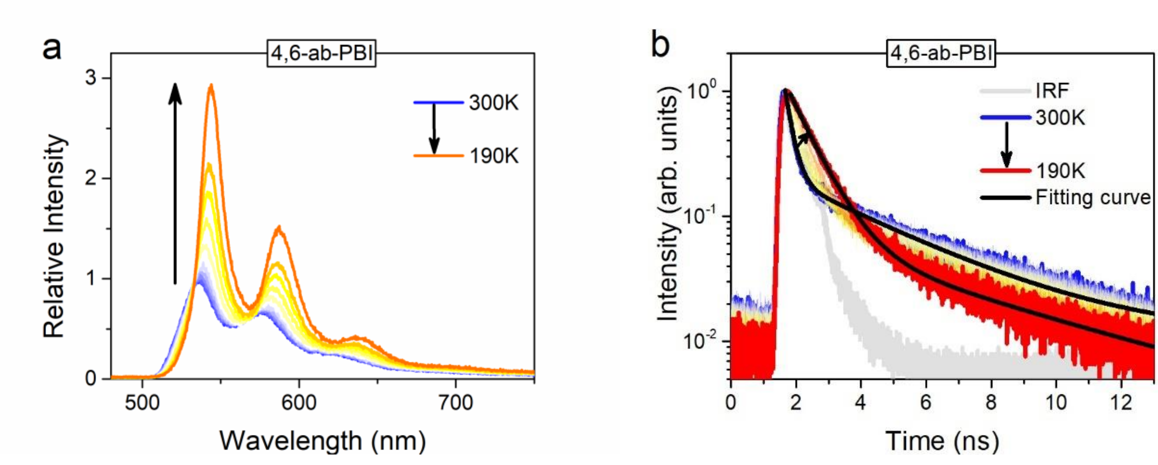

Supplementary Fig. 19 | a Temperature-dependent steady-state PL spectra (c = 10^{-7} M, λ_{ex}=480 nm) and **b** fluorescence decay of **4,6-ab-PBI** in chloroform solution.

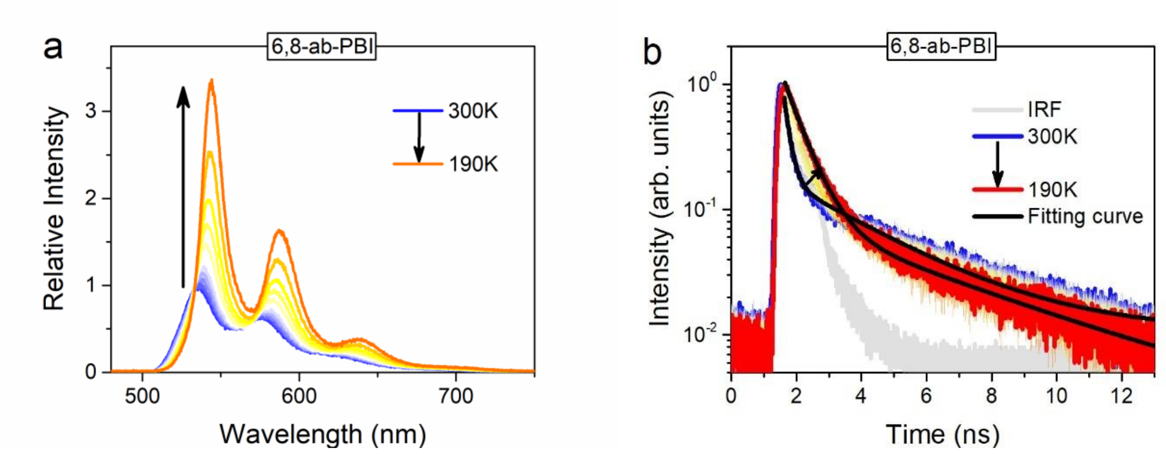

Supplementary Fig. 20 | a Temperature-dependent steady-state PL spectra (c = 10^{-7} M, λ_{ex}=480 nm) and **b** fluorescence decay of **6,8-ab-PBI** in chloroform solution; the IRF (in grey) is given for comparison. Solid lines are bi-exponential fits to the experimental data, obtained by deconvolution with the IRF.

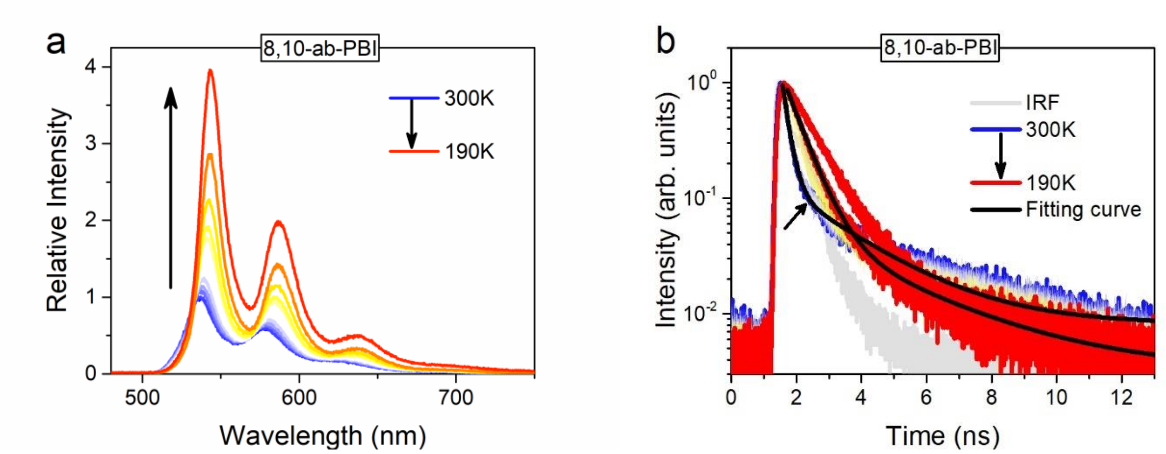

Supplementary Fig. 21 | a Temperature-dependent steady-state PL spectra (c = 10^{-7} M, λ_{ex}=480 nm) and **b** fluorescence decay of **8,10-ab-PBI** in chloroform solution; the IRF (in grey) is given for comparison. Solid lines are bi-exponential fits to the experimental data, obtained by deconvolution with the IRF.

Supplementary Table 7 | Lifetime of **4,6-ab-PBI, 6,8-ab-PBI and 8,10-ab-PBI** in chloroform solution at 300K and 190K. Freezing the conformational fluctuations could decrease the excitonic population of CT state. During the cooling process, the relative intensity becomes obviously stronger and the longer lifetime was observed.

PBI	300 K	190 K
4,6-ab-PBI	0.2 ± 0.001 (22%),<br>3.3 ± 0.03 (78%)	0.7 ± 0.003 (64%),<br>5.3 ± 0.4 (36%)
6,8-ab-PBI	0.2 ± 0.001 (24%),<br>2.9 ± 0.02 (76%)	0.6 ± 0.002 (61%),<br>4.5 ± 0.2 (39%)
8,10-ab-PBI	0.2 ± 0.001 (22%),<br>2.1 ± 0.02 (78%)	0.5 ± 0.001 (73%),<br>2.9 ± 0.1 (27%)

6. Photophysical Properties of the Thin Films

The structural characterization of annealing thin films was performed by X-ray scattering (GIWAXS) measurements with sample-detector distance (SDD) of 221.937 mm, which was determined by the first scattered peak at q = 0.107 Å-1 for silver behenate (AgBE). The incident angle was 0.2°, which was above the critical angle of most organic materials. Two images in vertical movement were combined to remove the horizontal gaps in the detector. The vertical sector integral was exported from the 2D GIWAXS pattern to give the structural information normal to the substrate, which indicated the molecular stacking orientation in the crystalline textured film based on the information of single crystal. The simulated equilibrium morphology showed the platelike geometric shapes (habits). As the prolongation of branched alkyl chains, the lamellar structure exhibited weaker interactions and the more obvious surface areas. In addition, the laminated structures could be fabricated on the silicon substrates by spin-coated and thermal annealing methods. The grazing-incidence wide-angle X-ray scattering (GIWAXS) patterns showed the (h00) reciprocal plane based on the information of single crystals, and the scattering signals in linear distribution along the out-of-plane direction indicated that the molecular lamellae were tiled layer by layer on the substrate. The first d-spacing were 2.99, 3.40 and 3.54 nm, which were attributed to the (100), (100) and (001) crystallographic planes for **4,6-ab-PBI**, **6,8-ab-PBI** and **8,10-ab-PBI** respectively. The pronounced lamellar structures demonstrate the molecular orientation: the geometric inclination angle of about 65° on the substrate for the three PBIs. Hence, the three dye compounds manifested the closely identical stacking modes on the molecular orientation and π-π contacts, but took on a tremendous discrepancy in fluorescence. In the conventional sense, strong π-π interactions were regarded as the prevalent factors on the radiative competitions, such as singlet fission, excimer trapping and symmetry-breaking charge separation, which produced multiple nonradiative channels and therefore caused fluorescence quenching. However, in this case, we observed the high fluorescence in the long-range π-stacked aggregate, which indicated that the π-stacked aggregate was luminous in nature when restricting the nonradiative access.

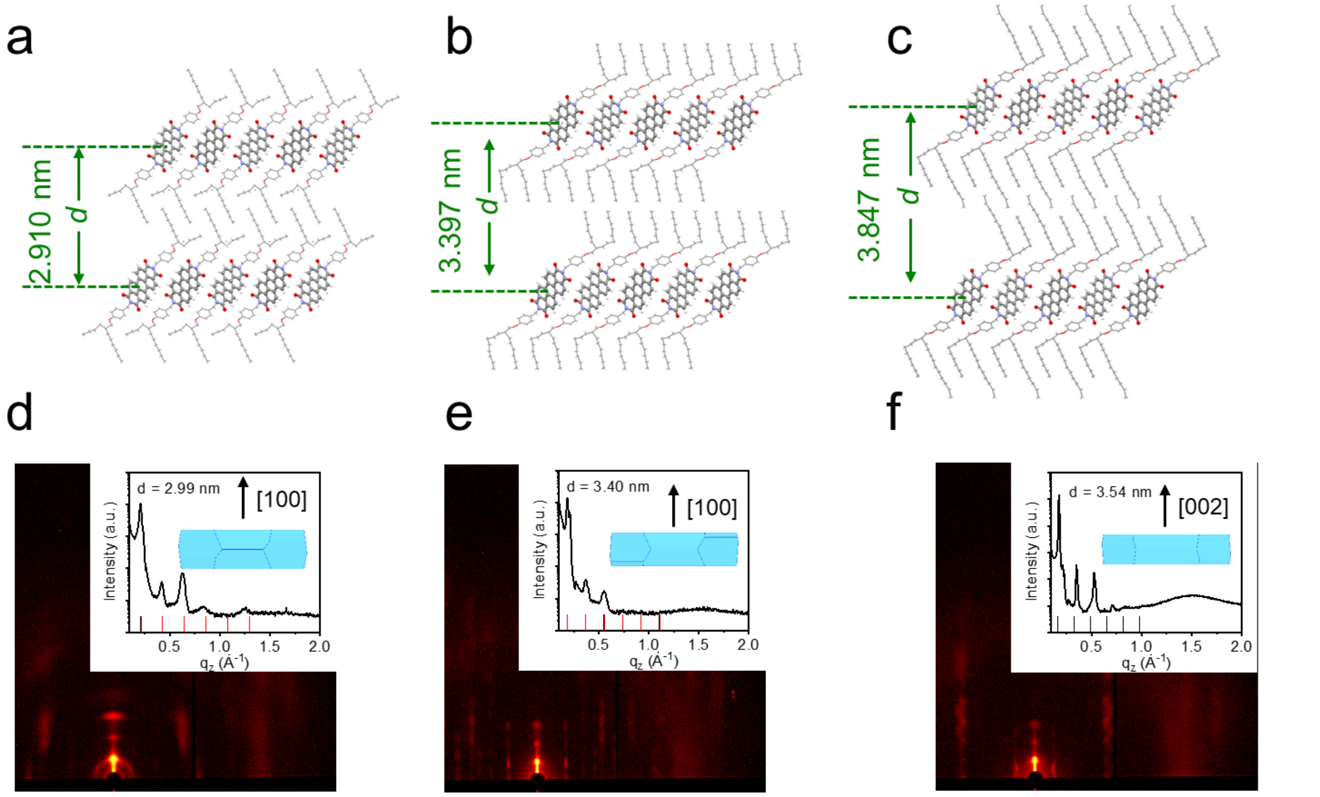

Supplementary Fig. 22 | Lamellar structure and interlamellar spacing of **a 4,6-ab-PBI** crystal, **b 6,8-ab-PBI** crystal, **c 8,10-ab-PBI** crystal. 2D GIWAXS patterns as well as 1D integral spectrum out of plane of **d** 4,6-ab-PBI, **e** 6,8-ab-PBI and **f** 8,10-ab-PBI polycrystalline films.

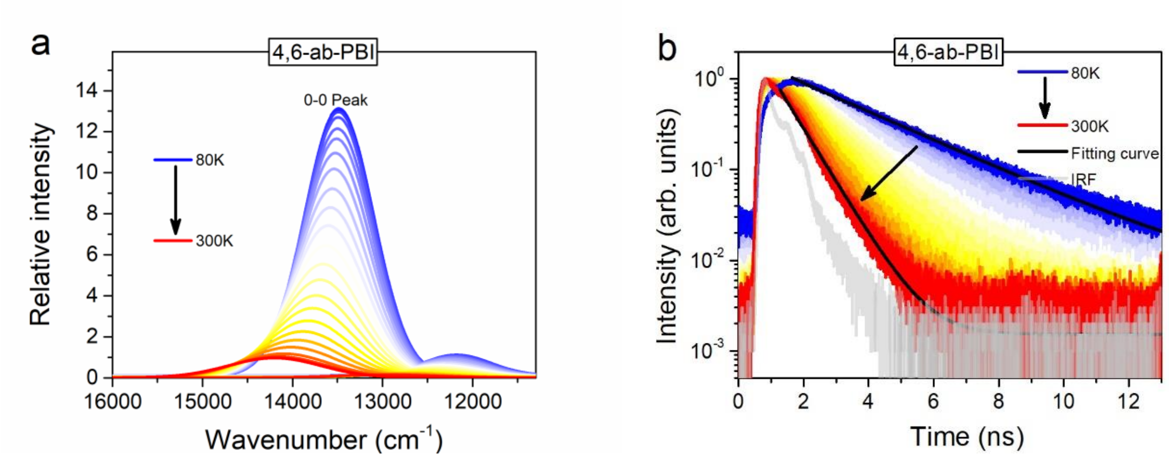

Supplementary Fig. 23 | Temperature-dependent **a** vibronic progression fitting from temperature-dependent PL spectra and **b** fluorescence decay of **4,6-ab-PBI** crystalline film; the IRF (in grey) is given for comparison. Solid lines are bi-exponential fits to the experimental data, obtained by deconvolution with the IRF.

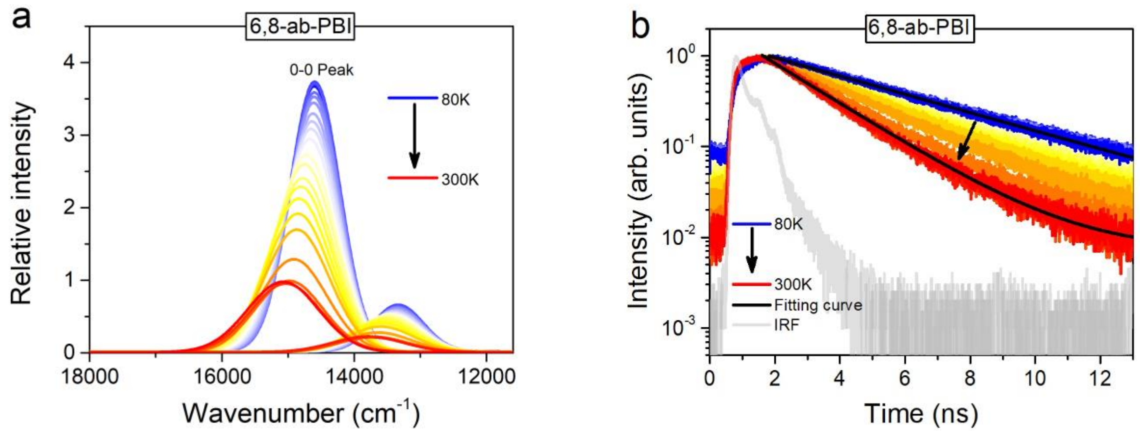

Supplementary Fig. 24 | Temperature-dependent **a** vibronic progression fitting from temperature-dependent PL spectra and **b** fluorescence decay of **6,8-ab-PBI** crystalline film; the IRF (in grey) is given for comparison. Solid lines are bi-exponential fits to the experimental data, obtained by deconvolution with the IRF.

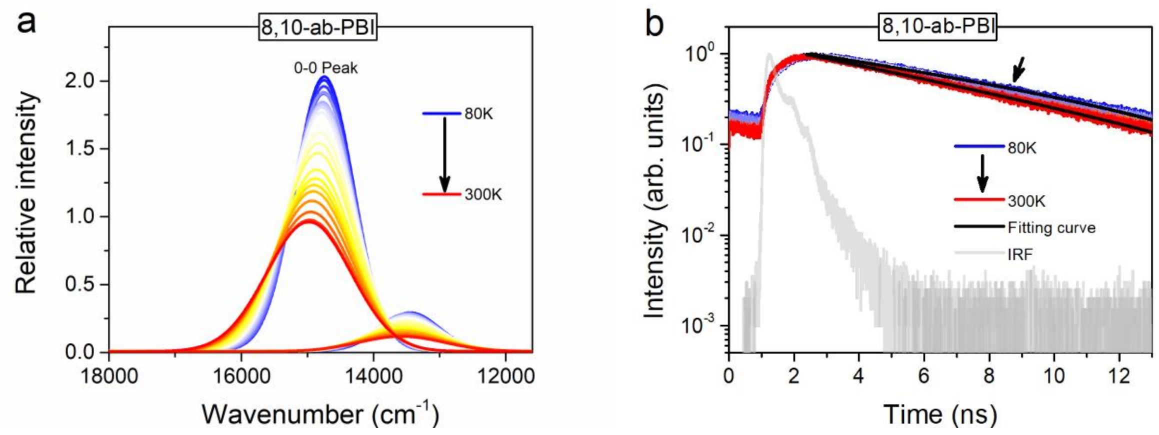

Supplementary Fig. 25 | Temperature-dependent **a** vibronic progression fitting from temperature-dependent PL spectra and **b** fluorescence decay of **8,10-ab-PBI** crystalline film; the IRF (in grey) is given for comparison. Solid lines are bi-exponential fits to the experimental data, obtained by deconvolution with the IRF.

Supplementary Table 8 | Lifetime of 4,6-ab-PBI, 6,8-ab-PBI and 8,10-ab-PBI polycrystalline films at 300K and 80K.

PBI	300 K	80 K
4,6-ab-PBI	0.7± 0.001<br>R^2=0.993	2.7 ± 0.01,<br>R^2=0.996
6,8-ab-PBI	1.9 ± 0.004,<br>R^2=0.997	4.3 ± 0.01,<br>R^2=0.996
8,10-ab-PBI	6.1 ± 0.03,<br>R^2=0.995	8.8 ± 0.07,<br>R^2=0.992

7. AIMD Simulation

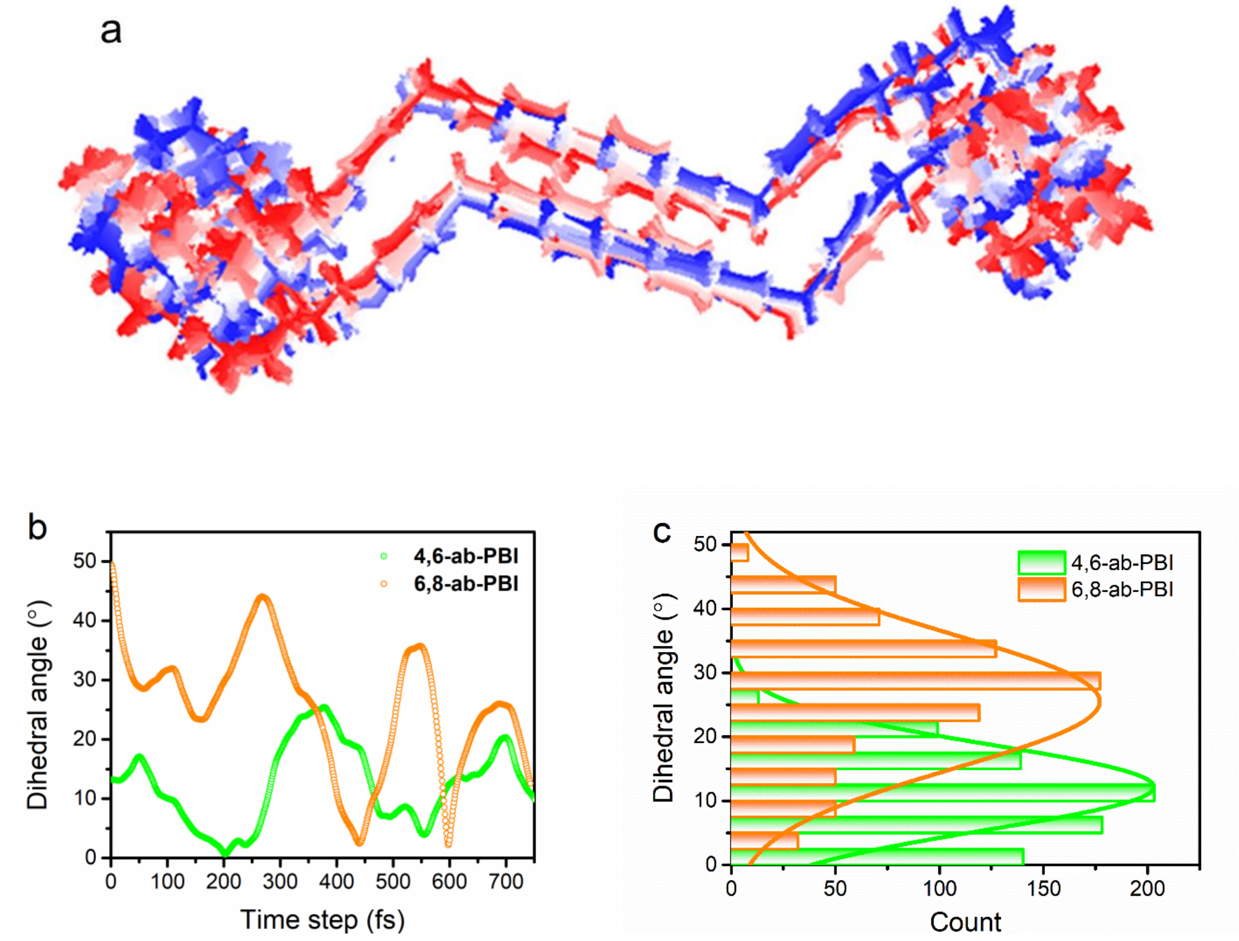

Supplementary Fig. 26 | (a) Trajectory of ab initio molecular dynamics simulation of 4,6-ab-PBI dimer in the time scale of 800 fs at room temperature. The structures are drawn every femtosecond, the color represents time step and varies as red-white-blue. (b) The quantitative statistics of dihedral angles on the trajectory at room temperature. (c) Normal distribution for 4,6-ab-PBI and 6,8-ab-PBI at room temperature. The relative motion between the adjacent chromophores was not evident, but the distribution of dihedral angles was relative to the length of alkyl chains rather than the temperature.

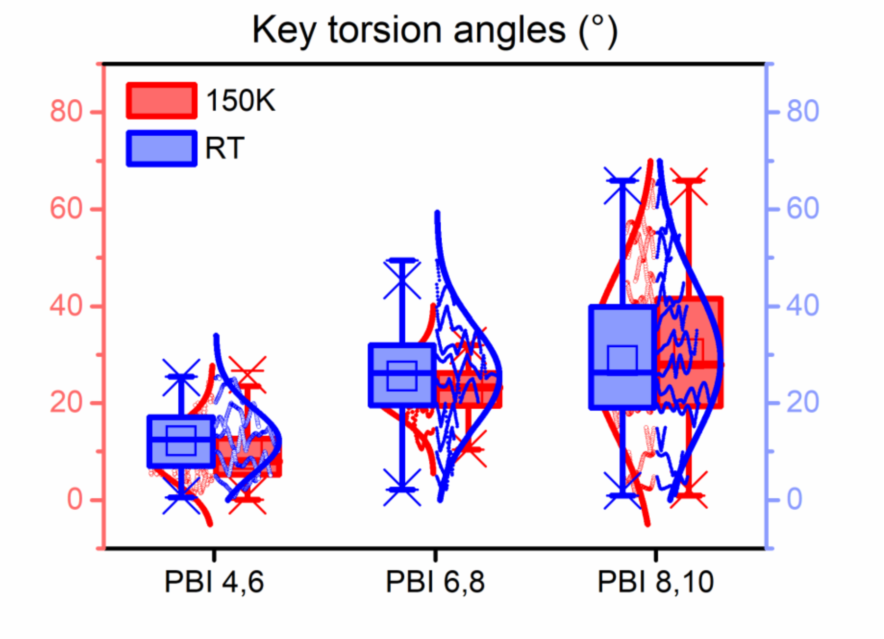

Supplementary Fig. 27 | The normal distribution of dihedral angles for **4,6-ab-PBI**, **6,8-ab-PBI** and **8,10-ab-PBI** at room temperature and 150K.

8. DFT Calculation

Supplementary Table 9 | The calculations were done in DCM using the PCM model, the B3LYP functional with the standard 6-311G* basis set was applied: vertical transition energies Evert for the first three singlet states with corresponding oscillator strength f and state descriptions (H=HOMO, L=LUMO). LE states are labelled in green, whereas CT states in red. In the in-plane case (0◦), the first two lowest excited states exhibit strong CT characters (S1: H-1→L 99 %, S2: H-2→L 98 %) with no oscillator strength, as dark states, they are responsible for the low quantum yield. The third excited state is a LE state (S3: H→L 99%) with a high oscillator strength (f=1.0147). As the dihedral angle increases, the amount of CT character at the phenoxyl group changes, hence the gap of vertical transition energies between CT and LE state becomes smaller, namely 0.09 eV in 4,6-ab-PBI and 0.02 eV in6,8-ab-PBI. On the other hand, LE is much little affected. Eventually, in an extreme out-of-plane case (90◦), a cross-over in the state ordering is observed, i. e. 2.28 eV for LE state and 2.68 eV for CT state.

ES Transitions	0°			PBI-4,6 (9°)			PBI-8,10 (26°)			PBI-6,8 (33°)			90°		
	E_{vert} (eV)	Osc. Str. f (a.u.)	MOs Transition (Contr. in %)	E_{vert} (eV)	Osc. Str. f (a.u.)	MOs Transition (Contr. in %)	E_{vert} (eV)	Osc. Str. f (a.u.)	MOs Transition (Contr. in %)	E_{vert} (eV)	Osc. Str. f (a.u.)	MOs Transition (Contr. in %)	E_{vert} (eV)	Osc. Str. f (a.u.)	MOs Transition (Contr. in %)
$S_0 \rightarrow S_1$	2.18	0.0000	H-1→L 99%	2.19	0.0000	H-1→L 99%	2.22	0.0576	H-2→L 88%	2.26	0.0000	H-1→L 99%	2.28	1.0220	H→ L 100%
$S_0 \rightarrow S_2$	2.18	0.0029	H-2→L 98%	2.19	0.0019	H-2→L 99%	2.22	0.000	H-1→L 99%	2.26	0.0007	H-2→L 99%	2.68	0.0000	H-1→L 99%
$S_0 \rightarrow S_3$	2.28	1.0147	H→L 99%	2.28	1.0167	H→L 100%	2.28	0.9677	H→L 89%	2.28	1.0202	H→L 100%	2.68	0.0003	H-2→L 99%

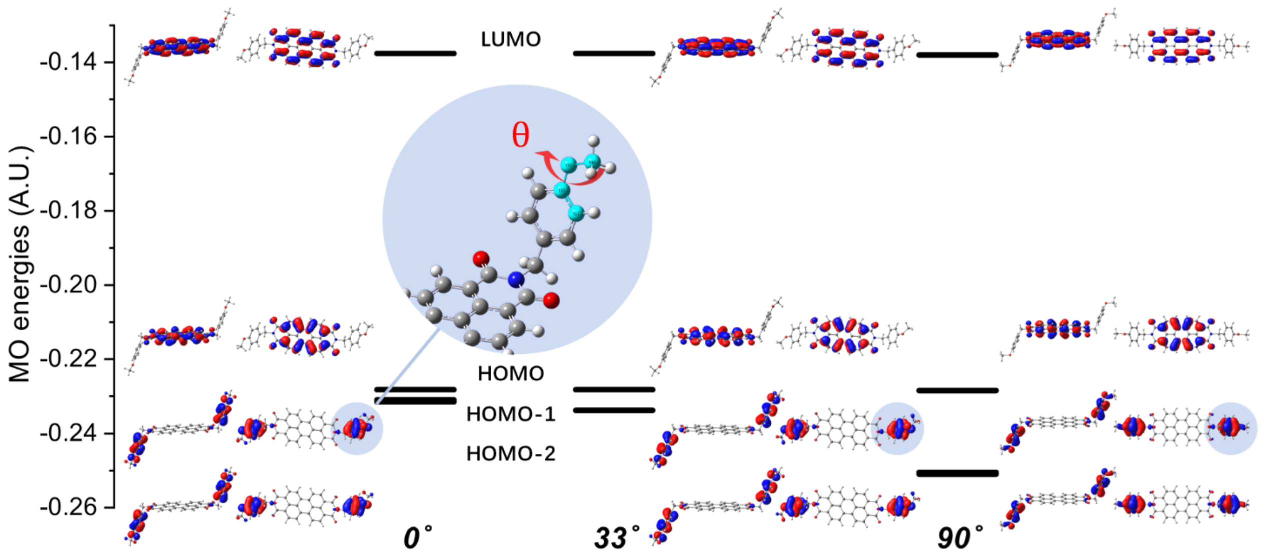

Supplementary Fig. 28 | Frontier MO energy levels and topologies for the different angles.

Supplementary Table 10 | Calculation results using the PCM model, CAM-B3LYP functional with the standard 6-311G* basis set.

ES Transitions	0°			PBI-4,6 (9°)			PBI-8,10 (26°)			PBI-6,8 (33°)			90°		
	E_{vert} (eV)	Osc. Str. f (a.u.)	MOs Transition (Contr. in %)	E_{vert} (eV)	Osc. Str. f (a.u.)	MOs Transition (Contr. in %)	E_{vert} (eV)	Osc. Str. f (a.u.)	MOs Transition (Contr. in %)	E_{vert} (eV)	Osc. Str. f (a.u.)	MOs Transition (Contr. in %)	E_{vert} (eV)	Osc. Str. f (a.u.)	MOs Transition (Contr. in %)
$S_0 \rightarrow S_1$	3.27	1.4041	H→L 90%	2.68	1.0855	H→L 98%	2.67	1.0890	H→L 98%	2.68	1.0871	H→L 98%	2.68	1.0877	H→L 98%
$S_0 \rightarrow S_2$	4.57	0.0000	H-5→L 66%<br>H→L+3 15%<br>H-6→L+1 7%	3.78	0.0000	H-1→L 87%	3.81	0.0000	H-5→L 50%<br>H-1→L 23%<br>H-8→L 9%<br>H→L+3 7%	3.81	0.0000	H-5→L 70%<br>H→L+3 11%<br>H-8→L 9%	3.81	0.0000	H-5→L 73%<br>H→L+3 12%<br>H-8→L 8%
$S_0 \rightarrow S_3$	4.69	0.0000	H→L+2 44%<br>H-8→L 41%	3.79	0.0000	H-2→L 91%	3.82	0.0000	H-1→L 63%<br>H-5→L 23%	3.85	0.0000	H-1→L 82%	4.00	0.0000	H-8→L 48%<br>H→L+2 28%<br>H-1→L 8%<br>H→L+3 6%
$S_0 \rightarrow S_4$	4.80	0.2492	H-6→L 68%<br>H→L+4 13%<br>H-5→L+1 9%	3.82	0.0000	H-5→L 73%<br>H→L+3 12%<br>H-8→L 7%	3.83	0.0000	H-2→L 91%	3.87	0.0001	H-2→L 90%	4.01	0.1051	H-6→L 78%<br>H→L+4 10%

Supplementary Table 11 | Calculation results using the PCM model, wB97XD functional with the standard 6-311G* basis set.

ES Transitions	0°			PBI-4,6 (9°)			PBI-8,10 (26°)			PBI-6,8 (33°)			90°		
	E_{vert} (eV)	Osc. Str. f (a.u.)	MOs Transition (Contr. in %)	E_{vert} (eV)	Osc. Str. f (a.u.)	MOs Transition (Contr. in %)	E_{vert} (eV)	Osc. Str. f (a.u.)	MOs Transition (Contr. in %)	E_{vert} (eV)	Osc. Str. f (a.u.)	MOs Transition (Contr. in %)	E_{vert} (eV)	Osc. Str. f (a.u.)	MOs Transition (Contr. in %)
$S_0 \rightarrow S_1$	2.72	1.0888	H→L 97%	2.72	1.0896	H→L 97%	2.71	1.0932	H→L 97%	2.72	1.0911	H→L 97%	2.71	1.0920	H→ L 100%
$S_0 \rightarrow S_2$	3.85	0.0000	H-5→L 69%<br>H→L+3 12%<br>H-7→L 10%	3.85	0.0000	H-5→L 69%<br>H→L+3 12%<br>H-7→L 10%	3.85	0.0000	H-5→L 70%<br>H→L+3 12%<br>H-7→L 9%	3.85	0.0000	H-5→L 69%<br>H→L+3 12%<br>H-7→L 10%	3.84	0.0000	H-5→L 70%<br>H→L+3 12%<br>H-7→L 9%
$S_0 \rightarrow S_3$	4.01	0.0000	H-7→L 37%<br>H-1→L 22%<br>H→L+2 21%	4.01	0.0000	H-7→L 38%<br>H→L+2 22%<br>H-1→L 21%	4.02	0.0000	H-7→L 39%<br>H→L+2 25%<br>H-1→L 13%	4.02	0.0000	H-7→L 43%<br>H→L+2 26%<br>H-1→L 11%<br>H-12→L 6%	4.03	0.1093	H-6→L 76%<br>H→L+4 11%<br>H-5→L+1 7%
$S_0 \rightarrow S_4$	4.02	0.0976	H-6→L 72%<br>H→L+4 10%<br>H-5→L+1 6%	4.03	0.0983	H-6→L 72%<br>H→L+4 10%<br>H-5→L+1 6%	4.03	0.1038	H-6→L 74%<br>H→L+4 10%<br>H-5→L+1 7%	4.03	0.1042	H-6→L 74%<br>H→L+4 11%<br>H-5→L+1 7%	4.04	0.0000	H-7→L 48%<br>H→L+2 30%<br>H→L+3 6%

Supplementary Table 12 | Calculation results using the PCM model, PBE0 functional with the standard 6-311G* basis set.

ES Transitions	0°			PBI-4,6 (9°)			PBI-8,10 (26°)			PBI-6,8 (33°)			90°		
	E_{vert} (eV)	Osc. Str. f (a.u.)	MOs Transition (Contr. in %)	E_{vert} (eV)	Osc. Str. f (a.u.)	MOs Transition (Contr. in %)	E_{vert} (eV)	Osc. Str. f (a.u.)	MOs Transition (Contr. in %)	E_{vert} (eV)	Osc. Str. f (a.u.)	MOs Transition (Contr. in %)	E_{vert} (eV)	Osc. Str. f (a.u.)	MOs Transition (Contr. in %)
$S_0 \rightarrow S_1$	2.37	0.9631	H→L 100%	2.37	0.9650	H→L 100%	2.37	0.9619	H→L 100%	2.37	0.9684	H→L 100%	2.37	0.9687	H→L 100%
$S_0 \rightarrow S_2$	2.46	0.0000	H-1→L 99%	2.46	0.0000	H-1→L 99%	2.50	0.0000	H-1→L 99%	2.54	0.0000	H-1→L 99%	2.97	0.0000	H-1→L 98%
$S_0 \rightarrow S_3$	2.46	0.0025	H-2→L 99%	2.46	0.0016	H-2→L 99%	2.50	0.0085	H-2→L 99%	2.54	0.0003	H-2→L 99%	2.98	0.0003	H-2→L 99%

Supplementary Table 13 | Calculation results using the PCM model, BHHLYP functional with the standard 6-311G* basis set.

ES Transitions	0°			PBI-4,6 (9°)			PBI-8,10 (26°)			PBI-6,8 (33°)			90°		
	E_{vert} (eV)	Osc. Str. f (a.u.)	MOs Transition (Contr. in %)	E_{vert} (eV)	Osc. Str. f (a.u.)	MOs Transition (Contr. in %)	E_{vert} (eV)	Osc. Str. f (a.u.)	MOs Transition (Contr. in %)	E_{vert} (eV)	Osc. Str. f (a.u.)	MOs Transition (Contr. in %)	E_{vert} (eV)	Osc. Str. f (a.u.)	MOs Transition (Contr. in %)
$S_0 \rightarrow S_1$	2.69	1.0916	H→L 98%	2.69	1.0928	H→L 98%	2.69	1.0947	H→L 98%	2.69	1.0947	H→L 98%	2.69	1.0956	H→L 98%
$S_0 \rightarrow S_2$	3.71	0.0000	H-1→L 95%	3.71	0.0000	H-1→L 95%	3.80	0.0000	H-1→L 95%	3.80	0.0000	H-1→L 94%	3.89	0.0000	H-5→L 78%<br>H→L+3 10%<br>H-8→L 7%
$S_0 \rightarrow S_3$	3.72	0.0002	H-2→L 96%	3.72	0.0002	H-2→L 96%	3.81	0.0001	H-2→L 96%	3.81	0.0001	H-2→L 96%	4.06	0.0000	H-8→L 36%<br>H-1→L 30%<br>H→L+2 24%

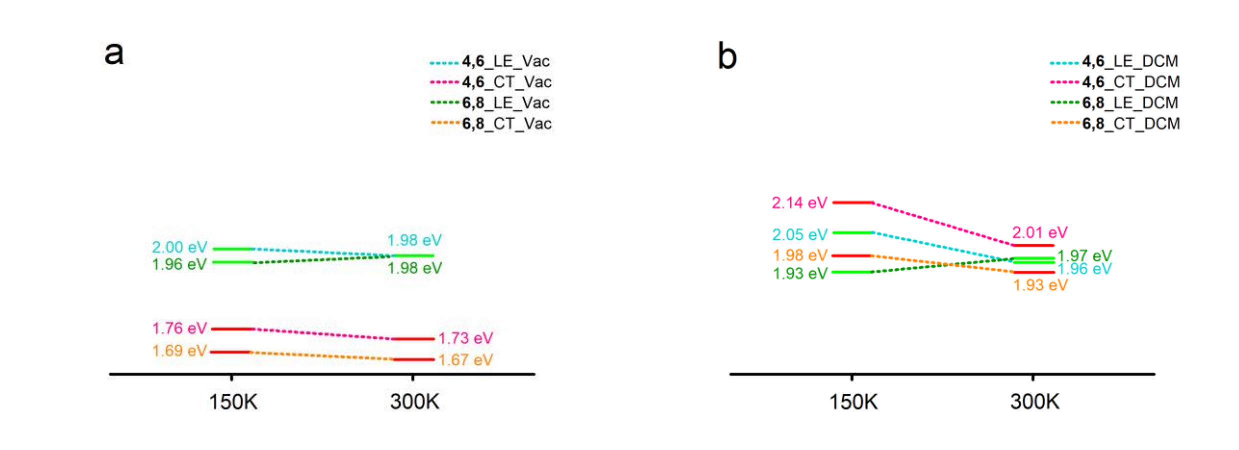

Supplementary Fig. 29 | Schematic diagram of the first representative transitions with LE and CT characters in (a) vacuum environment and (b) DCM environment. In vacuum, all lowest states with a CT character, even at room temperature. This can be concluded as a missing polarizability. Therefore, DCM was consequently introduced to compensate the description.

Supplementary Table 14 | Descriptions of the crystal structures and electronic transitions at 150 K and 300 K in DCM environment.

PBI	Temp.	Dihedral	DCM		ΔE
**4,6-ab-PBI** dimer	150K	8.6°/8.2°	CT<br>2.14 eV	LE<br>2.05 eV	0.09 eV
	300K	8.5°/8.5°	CT<br>2.01 eV	LE<br>1.96 eV	0.05 eV
**6,8-ab-PBI** dimer	150K	33.1°/33.1°	CT<br>1.98 eV	LE<br>1.93 eV	0.05 eV
	300K	4.0°/52.1°	CT<br>1.93 eV	LE<br>1.97 eV	0.04 eV

Supplementary Table 15| 4,6-ab-PBI ONIOM at 150 K in vacuum (8.6°/8.2°).

ES Transitions	E_{vert} (eV)	Osc. Str. f (a.u.)	MOs Transition	Contr. in %
S0->S1	1.76	0.0002	H -> L 41%, H -> L+1 23%<br>H-1 -> L+1 22%, H-1 -> L 13%	
S0->S2	1.76	0.0003	H-1 -> L 41%, H-1 -> L+1 23%<br>H -> L+1 22%, H -> L 13%	
S0->S3	1.85	0.0001	H -> L+1 43%, H-1 -> L 24%<br>H -> L 21%, H-1 -> L+1 11%	
S0->S4	1.85	0.0000	H-1 -> L+1 43%, H -> L 23%<br>H-1 -> L 21%, H -> L+1 11%	
S0->S5	1.98	0.0001	H-2 -> L+1 43%, H-2 -> L 41%<br>H-3 -> L 8%	
S0->S6	1.98	0.0004	H-3 -> L+1 44%, H-3 -> L 40%<br>H-2 -> L 7%	
S0->S7	2.00	0.0000	H-4 -> L+1 62%, H-5 -> L 24%<br>H-2 -> L+1 7%	
S0->S8	2.01	0.0261	H-4 -> L 39%, H-5 -> L+1 22%<br>H-2 -> L 15%, H-3 -> L 11%<br>H-3 -> L+1 7%, H-2 -> L+1 6%	
S0->S9	2.02	0.0004	H-2 -> L+1 27%, H-2 -> L 25%<br>H-3 -> L 23%, H-3 -> L+1 15%<br>H-4 -> L+1 7%	
S0->S10	2.02	0.0171	H-3 -> L+1 24%, H-4 -> L 23%<br>H-3 -> L 16%, H-5 -> L+1 13%<br>H-2 -> L+1 11%, H-2 -> L 11%	
S0->S11	2.29	0.0000	H-5 -> L 72%, H-4 -> L+1 27%	
S0->S12	2.46	1.3677	H-5 -> L+1 64%, H-4 -> L 36%	

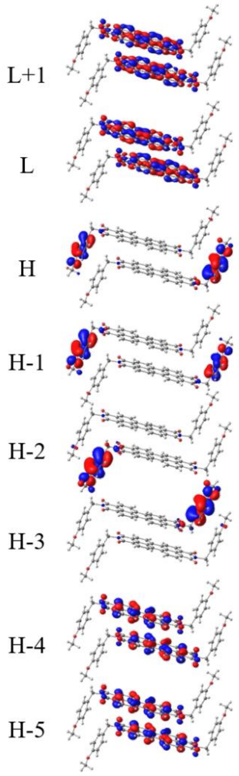

Supplementary Table 16 | 6,8-ab-PBI ONIOM at 150 K in vacuum (33.1°/33.1°).

ES Transitions	E_{vert} (eV)	Osc. Str. f (a.u.)	MOs Transition	Contr. in %
S0->S1	1.69	0.0004	H -> L 98%	
S0->S2	1.69	0.0003	H-1 -> L 98%	
S0->S3	1.81	0.0005	H -> L+1 97%	
S0->S4	1.82	0.0005	H-1 -> L+1 97%	
S0->S5	1.94	0.0003	H-2 -> L 83%<br>H-2 -> L+1 6%	
S0->S6	1.94	0.0008	H-3 -> L 75%<br>H-4 -> L 10%<br>H-3 -> L+1 5%	
S0->S7	1.96	0.0000	H-4 -> L 68%<br>H-3 -> L 13%<br>H-5 -> L+1 11%	
S0->S8	2.00	0.0009	H-5 -> L 50%<br>H-4 -> L+1 46%	
S0->S9	2.09	0.0002	H-2 -> L+1 87%<br>H-2 -> L 9%	
S0->S10	2.09	0.0003	H-3 -> L+1 87%<br>H-3 -> L 9%	
S0->S11	2.31	0.0000	H-5 -> L+1 84%<br>H-4 -> L 16%	
S0->S12	2.44	1.4112	H-4 -> L+1 50%<br>H-5 -> L 48%	

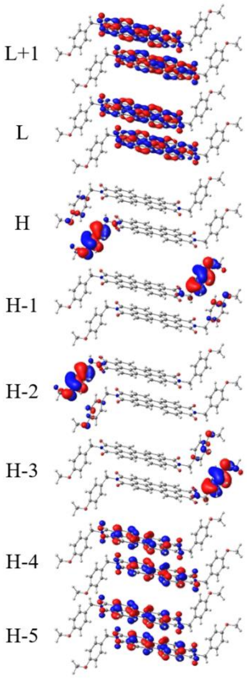

Supplementary Table 17 | 4,6-ab-PBI ONIOM at 150 K in DCM (8.6°/8.2°).

ES Transitions	E_{vert} (eV)	Osc. Str. f (a.u.)	MOs Transition	Contr. in %
**S0->S1**	**2.05**	**0.0000**	H -> L+1 73%, H-3 -> L 14%<br>H-5 -> L 10%	
S0->S2	2.06	0.1033	H -> L 69%, H-3 -> L+1 16%<br>H-5 -> L+1 13%	
S0->S3	2.14	0.0000	H-1 -> L 59%, H-2 -> L+1 38%	
S0->S4	2.14	0.0007	H-2 -> L 56%, H-1 -> L+1 41%	
S0->S5	2.23	0.0006	H-1 -> L+1 45%, H-2 -> L 31%<br>H-4 -> L 12%, H-3 -> L+1 6%	
S0->S6	2.23	0.0000	H-2 -> L+1 43%, H-1 -> L 30%<br>H-4 -> L+1 10%, H-3 -> L 9%	
S0->S7	2.27	0.0000	H-3 -> L 34%, H-4 -> L+1 24%<br>H-5 -> L 17%, H-2 -> L+1 15%<br>H-1 -> L 9%	
S0->S8	2.27	0.0021	H-4 -> L 51%, H-5 -> L+1 14%<br>H-1 -> L+1 13%, H-3 -> L+1 12%<br>H-2 -> L 9%	
S0->S9	2.29	0.0000	H-4 -> L+1 50%, H-3 -> L 40%<br>H -> L+1 9%	
S0->S10	2.29	0.0085	H-4 -> L 36%, H-3 -> L+1 33%<br>H-5 -> L+1 30%	
S0->S11	2.30	0.0000	H-5 -> L 67%, H-4 -> L+1 16%<br>H -> L+1 14%	
S0->S12	2.40	1.6593	H-5 -> L+1 39%, H-3 -> L+1 32%<br>H -> L 27%	

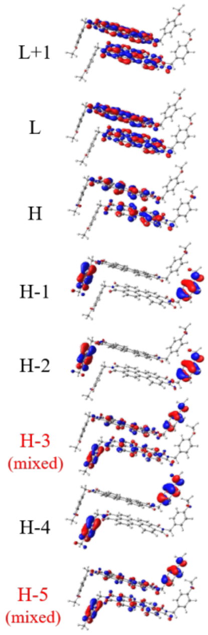

Supplementary Table 18 | 6,8-ab-PBI ONIOM at 150 K in DCM (33.1°/33.1°).

ES Transitions	E_{vert} (eV)	Osc. Str. f (a.u.)	MOs Transition	Contr. in %
**S0->S1**	**1.93**	**0.0000**	H-2 -> L 50%, H -> L 36% H-3 -> L+1 8%	
S0->S2	1.98	0.0015	H-1 -> L 63%, H-2 -> L+1 12% H-3 -> L 12%, H -> L 6%	
S0->S3	1.99	0.0000	H -> L 54%, H-2 -> L 39%	
S0->S4	1.99	0.0011	H-1 -> L 28%, H-3 -> L 24% H-2 -> L+1 20%, H -> L+1 12% H-5 -> L 9%	
S0->S5	2.10	0.0038	H -> L+1 37%, H-1 -> L+1 28% H-4 -> L 17%, H-3 -> L 8% H-2 -> L+1 7%	
S0->S6	2.10	0.0046	H-1 -> L+1 47%, H -> L+1 19% H-5 -> L 13%, H-2 -> L+1 11%	
S0->S7	2.15	0.0006	H-4 -> L 49%, H-3 -> L 14% H -> L+1 11%, H-1 -> L+1 11%	
S0->S8	2.16	0.0013	H-5 -> L 52%, H-4 -> L 11% H-1 -> L+1 11%, H-3 -> L 7% H-2 -> L+1 6%	
S0->S9	2.25	0.0000	H-3 -> L+1 78%, H-2 -> L 9%	
S0->S10	2.28	0.0111	H-4 -> L+1 82%	
S0->S11	2.29	0.0069	H-5 -> L+1 84%	
S0->S12	2.32	1.8046	H-2 -> L+1 39%, H-3 -> L 30% H-5 -> L 12%, H -> L+1 10% H-4 -> L 6%	

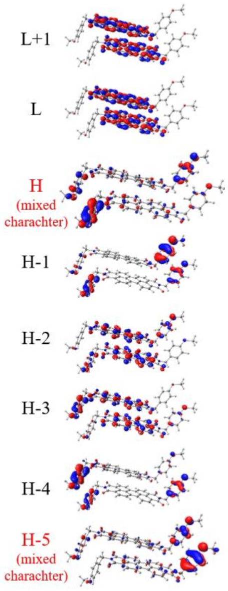

Supplementary Table 19 | 4,6-ab-PBI ONIOM at 300 K in vacuum (8.5°/8.5°).

ES Transitions	E_{vert} (eV)	Osc. Str. f (a.u.)	MOs Transition	Contr. in %
S0->S1	1.73	0.0004	H -> L 96%	
S0->S2	1.73	0.0000	H-1 -> L 96%	
S0->S3	1.80	0.0000	H-2 -> L 49% H -> L+1 39% H-1 -> L+1 4%	
S0->S4	1.81	0.0008	H-3 -> L 49% H-1 -> L+1 39%	
S0->S5	1.84	0.0000	H -> L+1 50% H-2 -> L 19% H-3 -> L 16% H-3 -> L+1 11%	
S0->S6	1.84	0.0001	H-1 -> L+1 51% H-3 -> L 19% H-2 -> L 16% H-2 -> L+1 11%	
S0->S7	1.94	0.0001	H-2 -> L+1 60% H-3 -> L+1 22% H-2 -> L 10%	
S0->S8	1.94	0.0001	H-3 -> L+1 59% H-2 -> L+1 23% H-3 -> L 10%	
S0->S9	1.98	0.0000	H-4 -> L 79% H-5 -> L+1 19%	
S0->S10	2.02	0.0017	H-4 -> L+1 51% H-5 -> L 48%	
S0->S11	2.31	0.0000	H-5 -> L+1 80% H-4 -> L 20%	
S0->S12	2.45	1.4624	H-5 -> L 51% H-4 -> L+1 48%	

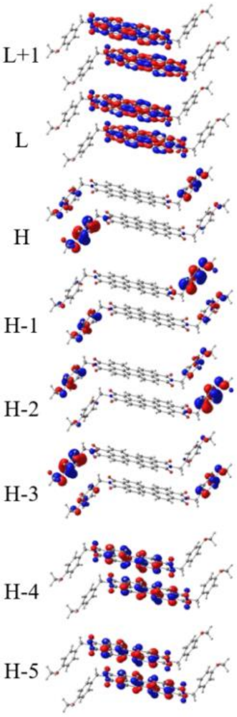

Supplementary Table 20 | 6,8-ab-PBI ONIOM at 300 K in vacuum (4.0°/52.1°).

ES Transitions	E_{vert} (eV)	Osc. Str. f (a.u.)	MOs Transition	Contr. in %
S0->S1	1.67	0.0007	H -> L 97%	
S0->S2	1.75	0.0013	H -> L+1 96%	
S0->S3	1.88	0.0014	H-1 -> L 86%<br>H-1 -> L+1 13%	
S0->S4	1.95	0.0002	H-2 -> L 90%<br>H-3 -> L 6%	
S0->S5	1.98	0.0002	H-4 -> L 62%<br>H-5 -> L+1 15%<br>H-3 -> L 9%	
S0->S6	2.00	0.0006	H-1 -> L+1 62%<br>H-5 -> L 13%<br>H-4 -> L+1 13%<br>H-1 -> L 8%	
S0->S7	2.01	0.0016	H-2 -> L+1 47%<br>H-3 -> L 41%<br>H-4 -> L 6%	
S0->S8	2.03	0.0009	H-4 -> L+1 29%<br>H-5 -> L 27%<br>H-1 -> L+1 21%<br>H-4 -> L 9%<br>H-5 -> L+1 8%	
S0->S9	2.07	0.0001	H-2 -> L+1 44%<br>H-3 -> L 31%<br>H-3 -> L+1 20%	
S0->S10	2.13	0.0001	H-3 -> L+1 74%<br>H-3 -> L 10%<br>H-2 -> L+1 6%<br>H-2 -> L 6%	
S0->S11	2.30	0.0001	H-5 -> L+1 75%<br>H-4 -> L 22%	
S0->S12	2.44	1.4513	H-5 -> L 49%<br>H-4 -> L+1 49%	

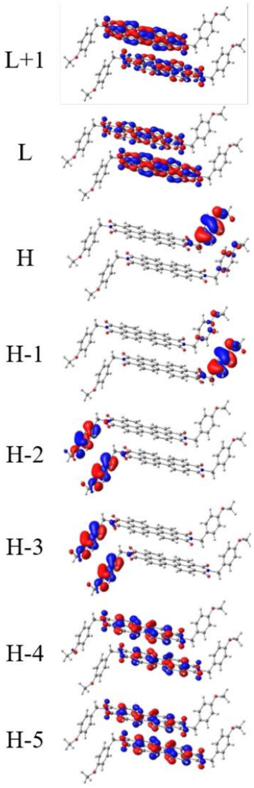

Supplementary Table 21 | 4,6-ab-PBI ONIOM at 300 K in DCM (8.5°/8.5°).

ES Transitions	E_{vert} (eV)	Osc. Str. f (a.u.)	MOs Transition	Contr. in %
S0>S1	1.96	0.0000	H-2 -> L 78%, H-5 -> L+1 15%<br>H-1 -> L 5%	
S0>S2	2.00	0.0018	H-2 -> L+1 39%, H-5 -> L 32%<br>H -> L 22%, H-3 -> L 5%	
S0>S3	2.01	0.0000	H-1 -> L 84%, H-2 -> L 6%	
S0>S4	2.01	0.0019	H -> L 69%, H-5 -> L 13%<br>H-2 -> L+1 9%	
S0>S5	2.06	0.0001	H-4 -> L 65%, H -> L+1 28%	
S0>S6	2.06	0.0036	H-3 -> L 63%, H-1 -> L+1 28%	
S0>S7	2.12	0.0000	H -> L+1 61%, H-4 -> L 18%<br>H-3 -> L+1 15%	
S0>S8	2.12	0.0005	H-1 -> L+1 55%, H-3 -> L 19%<br>H-4 -> L+1 15%, H-2 -> L+1 7%	
S0>S9	2.20	0.0004	H-3 -> L+1 60%, H-4 -> L+1 18%<br>H-4 -> L 7%, H -> L+1 6%	
S0>S10	2.20	0.0009	H-4 -> L+1 59%, H-3 -> L+1 18%<br>H-1 -> L+1 7%, H-3 -> L 6%	
S0>S11	2.25	0.0000	H-5 -> L+1 85%, H-2 -> L 14%	
S0>S12	2.33	1.8572	H-5 -> L 51%, H-2 -> L+1 44%	

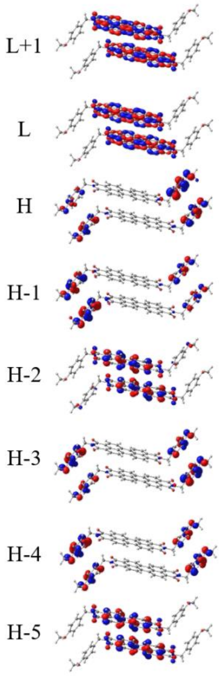

Supplementary Table 22 | 6,8-ab-PBI ONIOM at 300 K in DCM (4.0°/52.1°).

ES Transitions	E_{vert} (eV)	Osc. Str. f (a.u.)	MOs Transition	Contr. in %
S0->S1	1.93	0.0008	H -> L 94%	
S0->S2	1.97	0.0005	H-1 -> L 67%, H-3 -> L+1 15% H-2 -> L 8%	
S0->S3	2.00	0.0033	H-3 -> L 38%, H-1 -> L+1 30% H -> L+1 21%, H-2 -> L+1 6%	
S0->S4	2.01	0.0054	H -> L+1 61%, H-2 -> L 23% H-1 -> L+1 8%	
S0->S5	2.08	0.0025	H-2 -> L 47%, H-1 -> L 13% H -> L+1 12%, H-1 -> L+1 11% H-2 -> L+1 8%, H-3 -> L 8%	
S0->S6	2.18	0.0001	H-2 -> L+1 69%, H-2 -> L 14% H-1 -> L+1 11%	
S0->S7	2.20	0.0006	H-4 -> L 89%	
S0->S8	2.24	0.0001	H-3 -> L+1 80%, H-1 -> L 15%	
S0->S9	2.25	0.0368	H-5 -> L 55%, H-4 -> L+1 40%	
S0->S10	2.32	0.0984	H-4 -> L+1 48%, H-5 -> L 26% H-5 -> L+1 18%	
S0->S11	2.33	1.6993	H-3 -> L 43%, H-1 -> L+1 36% H-5 -> L 9%, H-2 -> L+1 9%	

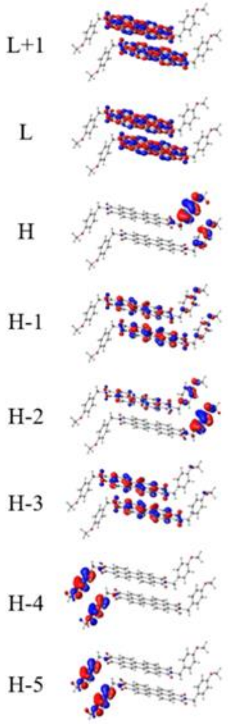

9. Supplementary References

1. Dolomanov, O.V. et al. OLEX2: a complete structure solution, refinement and analysis program. *J. Appl. Cryst.* **42**, 339-341 (2009).
2. Sheldrick, G. M. SHELXT: Integrating space group determination and structure solution. *Acta Crystallogr. A*, **70**, C1437 (2014).
3. Sheldrick, G. M. SHELXT - Integrated space-group and crystal-structure determination. *Acta Crystallogr. A*, **71**, 3-8 (2015).
4. Lee. C., Yang, W. & Parr, R. G. Development of the Colle-Salvetti correlation-energy formula into a functional of the electron density. *Phys. Rev. B* **37**, 785-789 (1988).
5. Becke, A. D. Density-functional thermochemistry. III. The role of exact exchange. *J. Chem. Phys.* **98**, 5648-5652 (1993).
6. Hehre, W. J., Ditchfield, R. & Pople, J. A. Self-consistent molecular orbital methods. XII. Further extensions of Gaussian-type basis sets for use in molecular orbital studies of organic molecules. *J. Chem. Phys.* **56**, 2257-2261 (1972).
7. Francl, M. M. et al. Self-consistent molecular orbital methods. XXIII. A polarization-type basis set for second-row elements. *J. Chem. Phys.* **77**, 3654-3665 (1982).
8. Harcourt, R. D., Ghiggino, K. P., Scholes, G. D. & Speiser, S. On the origin of matrix elements for electronic excitation (energy) transfer. *J. Chem. Phys.* **105**, 1897-1901(1996).

9. Yamagata, H., Pochas, C. M. & Spano, F. C. Designing J- and H-Aggregates through wave function overlap engineering: applications to poly(3-hexylthiophene). *J. Phys. Chem. B* **116**, 14494-14503 (2012).

10. Hestand, N. J. & Spano, F. C. Interference between Coulombic and CT-mediated couplings in molecular aggregates: H- to J-aggregate transformation in perylene-based pi-stacks. *J. Chem. Phys.* **143**, 244707 (2015).

11. Li, X. Y., Tang, X. S. & He, F. C. Electron transfer in poly(p-phenylene) oligomers: effect of external electric field and application of Koopmans theorem. *Chem. Phys.* **248**, 137-46 (1999).

12. Yamagata, H. et al. HJ-Aggregate Behavior of Crystalline 7,8,15,16-Tetraazaterrylene: Introducing a New Design Paradigm for Organic Materials. *J. Phys. Chem. C* **118**, 28842-28854 (2014).

13. Madjet, M. E., Abdurahman, A. & Renger, T. Intermolecular Coulomb couplings from ab initio electrostatic potentials: Application to optical transitions of strongly coupled pigments in photosynthetic antennae and reaction centers. *J. Phys. Chem. B* **110**, 17268-17281 (2006).

14. Shen, Z. & Forrest, S. R. Quantum size effects of charge-transfer excitons in nonpolar molecular organic thin films. *Phys. Rev. B*, **55**, 10578-10592 (1997).
